# Prenatal exposure to phthalate and decreased body mass index of children: a systematic review and meta-analysis

**DOI:** 10.1038/s41598-022-13154-9

**Published:** 2022-05-27

**Authors:** Dong-Wook Lee, Hyun-Mook Lim, Joong-Yub Lee, Kyung-Bok Min, Choong-Ho Shin, Young-Ah Lee, Yun-Chul Hong

**Affiliations:** 1 Public Healthcare Center, Seoul National University Hospital, 101 Daehak-ro, Jongno-gu, Seoul, 03080 Republic of Korea; 2 Department of Preventive Medicine, Seoul National University College of Medicine, 103 Daehak-ro, Jongno-gu, Seoul, 03080 Republic of Korea; 3 Department of Pediatrics, Seoul National University College of Medicine, 103 Daehak-ro, Jongno-gu, Seoul, 03080 Republic of Korea; 4 Department of Humans Systems Medicine, Seoul National University College of Medicine, 103 Daehak-ro, Jongno-gu, Seoul, 03080 Republic of Korea; 5 COMWEL Daejeon Hospital, Korea Workers’ Compensation & Welfare Service, Daejeon, Republic of Korea

**Keywords:** Environmental sciences, Endocrinology, Risk factors

## Abstract

Phthalates are well-known endocrine-disrupting chemicals. Many detrimental health effects of phthalates were investigated, but studies on the association of phthalates with obesity in children showed inconsistent results. Thus, this systematic review and meta-analysis were performed to clarify whether prenatal and postnatal exposures to phthalates are associated with physical growth disturbances in children. We performed the systematic review and meta-analysis following the PRISMA 2020 statement guidelines, and found 39 studies that met our inclusion criteria, including 22 longitudinal and 17 cross-sectional studies. We observed a significant negative association between the prenatal exposure to DEHP and the body mass index (BMI) z-score of the offspring (β = − 0.05; 95% CI: − 0.10, − 0.001) in the meta-analysis, while no significant association between the prenatal exposure to DEHP and the body fat percentage of the offspring was observed (β = 0.01; 95% CI: − 0.41, 0.44). In the systematic review, studies on the association between phthalates exposure in childhood and obesity were inconsistent. Prenatal exposure to phthalates was found to be associated with decreased BMI z-score in children, but not associated with body fat percentage. Our findings suggest that phthalates disturb the normal muscle growth of children, rather than induce obesity, as previous studies have hypothesized.

## Introduction

Phthalates are wildly used chemicals to improve the utility of plastics and personal care products. Due to phthalates’ low price and usefulness, the annual global production of phthalates is estimated up to 5.5 million tonnes^1^. Phthalates can be classified as high-molecular-weight phthalates (HMWPs) and low-molecular-weight phthalates (LMWPs). HMWPs can give plastics flexibility, and were used in toys, building materials, medical devices, and paints. Di-(2-ethylhexyl) phthalate (DEHP), the most widely used HMWP, accounts for 65.2% of the total consumption of phthalates, and is produced approximately 2 million tonnes per year^2,3^. Meanwhile, LMWPs are usually used in cosmetics such as shampoos, cosmetics, lotions, nail care products, and other personal hygiene products; dibutyl phthalate (DBP) is one of the most widely used LMWPs^4^.

Phthalate is a well-known endocrine-disrupting chemical with anti-androgenic effects. Due to its anti-androgenic properties, previous studies have focused on the health effects of phthalates, including abnormal sexual development such as hypospadias and anogenital distance, adverse birth outcomes, precocious puberty, and hormonal disturbances of testosterone and thyroid hormone^5–9^. However, the relationship between phthalates and obesity remains unclear and inconclusive, although phthalates can interfere with growth and metabolism^10–14^.

Recently, the negative association between the perinatal exposure to phthalates and the body weight was reported in a systematic review and meta-analysis from animal studies^15^. A rodent study reported that prenatal exposure to DEHP could induce decreased muscle mass^16^. Although a recent systematic review and meta-analysis of human studies was performed, a definitive conclusion in children was not reached^17^. Considering these evidence, the hypothesis that phthalates exposure is associated with obesity should be revised as phthalate exposure are associated with disturbing normal growth. Therefore, it is necessary to review and summarize the direction and size of associations found in the studies using the latest results.

The present study aimed to clarify whether prenatal and postnatal exposures to phthalates are associated with physical growth as measured by body composition indices in children. Thus, we performed a systematic literature review and meta-analysis for the association of phthalates with body composition indices among children.

## Materials and methods

### Search strategy and selection methods

This study was registered in PROSPERO, a prospective international register of systematic reviews (CRD42021235007). The review question was as follows: “Does the prenatal and postnatal exposures to phthalates affect the physical growth of children?” According to PECO formulation guidance^18^, more specifically, the review question was “among the children, what is the effect of one-unit of natural log of DEHP (or DBP) metabolites versus one-unit incremental increase on the physical growth measured by BMI, body fat percentage, and other indices”. We used PubMed, EMBASE, and Google Scholar to search articles that reported associations between the DEHP and DBP levels and the physical growth of children between January 1, 1980, and December 31, 2021, using the search string (Supplementary Table S1). The inclusion criteria were: (1) the epidemiologic study in a cohort, case–control, or cross-sectional design; and (2) the size of association reported in beta estimates (β) with 95% confidence intervals (CIs), or in the form that can be converted to β and 95% CIs. The exclusion criteria were: (1) presented outcomes in irrelevant forms; (2) not able to use the size of the association; (3) letter, commentary, or review articles; (4) investigated the identical study population to other included study; (5) articles not written in English; and (6) non-human.

Following the PRISMA statement guidelines for reporting systematic reviews and meta-analysis^19^, we systematically searched the literature databases. The search results of each search were downloaded into a reference management software program (EndNote, version X8) for identifying duplicate articles and for further review. Two authors (DW-L and HM-L) screened records and selected articles according to the inclusion and exclusion criteria. If two authors disagreed about eligibility of a study, the authors agreed after discussion and understanding with a third author (YC-H). Finally, the authors manually checked the reference lists of the included articles.

### Data extraction

We extracted the following data from all articles using a data-extraction sheet: first author, year, country, type of study, sample size, the timing of exposure assessment, measured metabolites and the corresponding range, statistical analysis, adjustment variables, timing of outcome assessment, outcome variables, findings, estimates type (β estimate and/or odds ratio [OR]), and estimates and 95% CIs of the association between prenatal phthalate exposure and outcome variables.

The classes of DEHP metabolites were measured not identically across studies. Included studies reported the size of associations of outcome variables with each DEHP metabolite, with or without their sum (∑DEHP). We preferred the size of association of outcome variables with ∑DEHP as an exposure indicator for our meta-analysis. Among secondary metabolites, the available metabolites were selected in the following order according to the molar fraction of excretion to absorbed DEHP in the human body: mono-2-ethyl-5-carboxypentyl phthalate (MECCP), mono-(2-ethyl-5-hydroxy-hexyl) phthalate (MEHHP), and mono-(2-ethyl-5-oxo-hexyl) phthalate (MEOHP)^20^. Among the DBP metabolites, mono-n-butyl phthalate (MnBP) was preferentially selected, followed by mono-isobutyl phthalate (MiBP)^21^. If it was not available to use the size of association of secondary metabolites of DEHP, mono-(2-ethylhexyl) phthalate was used as an indicator of exposure. In each study, we used β estimates with 95% CIs with the model with the most adjustment variables was used for meta-analysis.

### Quality assessment

The Newcastle–Ottawa quality assessment scale (NOS) was used^22^. The NOS for cohort and cross-sectional studies consists of selection, comparability, and outcome assessment items. By using the assessment tool, the quality of cohort studies was scored from 0 to 9 and classified as low (0–3), moderate (4–6), or high (7–9). The quality of cross-sectional studies was scored from 0 to 10 and classified into low (0–3), moderate (4–7), or high (8–10).

### Statistical analysis

Four meta-analyses for prenatal exposure and body composition in children were performed according to the phthalates (DEHP or DBP) × outcome (body mass index [BMI] z-score and body fat percentage). For the studies on the association between postnatal exposure and body composition in children, we could not perform a meta-analysis for the heterogeneity in the age of study participants, measured outcome, and presented the size of the association. The standardized regression coefficient for effect size and its standard error were used for meta-analyses of the association between phthalates and body composition^23^. The heterogeneity of results across studies was examined by Q test, with *P* < 0.10 implying substantial heterogeneity. The overall estimate was calculated using a random-effects model, considering the between-study variation. We evaluated publication bias by a Begg funnel plot and the Egger test. If the asymmetry of the funnel plot, and/or *P* < 0.05 from the Egger test were found, we considered the existence of publication bias. Statistical analyses were conducted using the R software, “metafor” package, version 2.13.2 (Wolfgang Viechtbauer, Maastricht, the Netherlands).

### Ethics approval and consent to participate

Not applicable (no human subject participants will be involved).

### Consent for publication

Not applicable.

## Results

**Figure 1:**
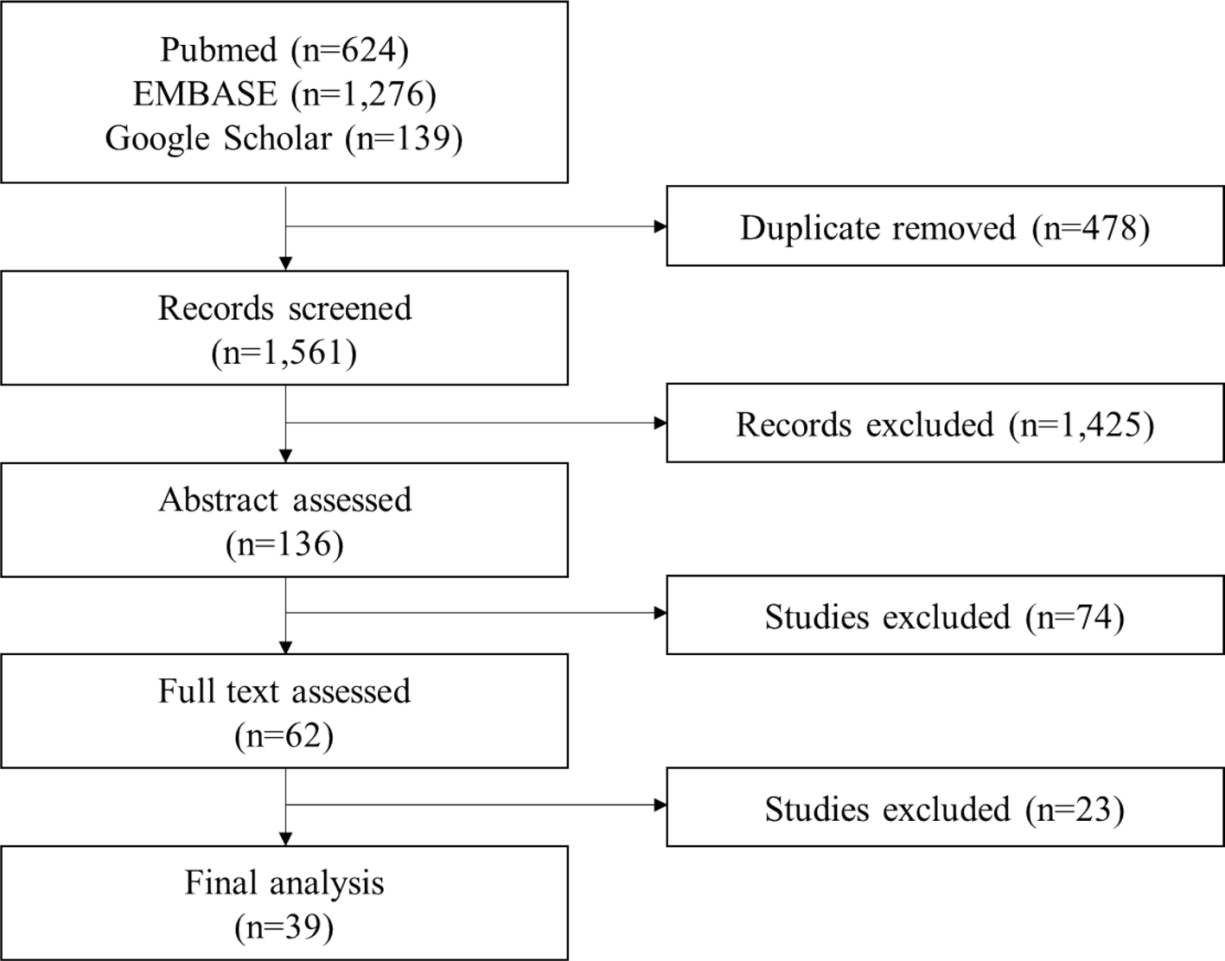
Flow diagram of the study selection process.

Figure 1 shows the process to include the relevant studies for the systematic review. We screened 1561 records, and excluded 1425 studies based on their titles. After the abstracts were reviewed, 74 irrelevant studies were excluded. The full texts of 62 studies were assessed, and we found 39 studies that met our inclusion criteria. The reference lists of the included studies were manually checked, and no additional studies were searched in this step.

Table 1 summarizes the a total of 39 observational studies. The study size varied between 72 and 2,884 participants. Studies were conducted in the United States (n = 12), China (n = 6), South Korea (n = 4), Taiwan (n = 3), Spain (n = 2), Netherlands (n = 1), Australia (n = 1), France (n = 1), Greece (n = 1), Italy (n = 1), Germany (n = 1), Canada (n = 1), Sweden (n = 1), Thailand (n = 1), Iran (n = 1), Mexico (n = 1), and in multiple European countries (n = 1). Supplementary Table S2 shows reasons for exclusion in full-text review. The quality of the studies as assessed by the NOS is presented in Supplementary Table S3–4. The scores of the included longitudinal studies (n = 22) ranged from 8 to 9, and all were classified as good quality. Cross-sectional studies (n = 17) ranged from 5 to 8 and included 12 high-quality studies and 5 moderate-quality studies.

**Table 1:** Summary of studies included in the systematic review.

ID	First author	Year	Study design	Country	Sample Size	Study	Exposure assessment	Measured metabolites and range	Timing of outcome assessment	Outcome variables
1	Agay-Shay^24^	2015	Cohort study	Spain	470	INMA Spanish Birth cohort	Maternal urine in the 1st and 3rd trimester of pregnancy	GM of MECPP, MEHHP, MEOHP, and MEHP (40.8 µg/g Cr, 28.6 µg/g Cr, 27.8 µg/g Cr, and 14.6 µg/g Cr, respectively) ‘GM of MnBP and MiBP (32.4 µg/g Cr, 32.6 µg/g Cr, respectively)	7 y	BMI z-scores
2	Berman^25^	2020	Cohort study	Australia	410		Maternal urine in the 2nd and 3rd trimester of pregnancy	Median of ∑DEHP metabolites, and ∑DBP metabolites (9.34 µg/L, 4.10 µg/L, respectively)	1, 2, 3, 5, 8, 10, 14, 17 and 20 y	Height, BMI, DXA (total fat %, total fat mass [g], total lean mass [g])
3	Botton^26^	2016	Cohort study	France	520	EDEN mother–child cohort	Maternal urine in the 2nd trimester	Median of molar ∑DEHP metabolites, MnBP and MiBP (0.32 µM/L, 43 µg/L, and 39 µg/L, respectively)	5 y	BMI
4	Buckley^27^	2016	Cohort study	U.S	707	MSSM + CCCEH + HOME Study	Prenatal maternal urine	GM of molar ∑DEHP metabolites, MnBP and MiBP (0.277 µM/L, 30.6 µg/L, and 6.45 µg/L, respectively)	4–9 y	BMI z-score and overweight/obese (BMI > = 85th percentile)
5	Buckley^10^	2016	Cohort study	U.S	180	MSSM Study	Prenatal maternal urine	GM of molar ∑DEHP metabolites, MnBP and MiBP (0.284 µM/L, 32.9 µg/L, and 5.83 µg/L, respectively)	4 and 9 y	Body composition (total fat %)
6	Buser^28^	2014	Cross-sectional study	U.S	not described	NHANES 2007–2010	Urine of the participants	(Children and adolescent aged 6–19) GM of molar ∑DEHP metabolites, MnBP and MiBP (0.24 µM/L, 23.0 µg/L, and 10.43 µg/L, respectively) (adults > = 20 y) GM of ∑DEHP metabolites, MnBP and MiBP (0.18 µM/L, 15.21 µg/L, and 6.75 µg/L, respectively)	Children and adolescent aged 6–19, adults > = 20 y	(Children and adolescent) obese 95^th^ percentile > = BMI z-score; overweight, 95^th^ percentile > BMI z-score > = 85^th^ percentile (adults) obese, BMI > = 30 kg/m^2^; overweight, 30 kg/m^2^ > BMI > = 25 kg/m^2^
7	Chang^29^	2020	Cross-sectional study	Taiwan	152	RAPIT program	Urine of the participants	GM of ∑DEHP metabolites, MnBP and MiBP (59.29 µg/g Cr, 49.44 µg/g Cr, and 28.85 µg/g Cr, respectively)	5 y	BMI, total fat (%)
8	Deierlein^30^	2016	Cohort study	U.S	1,239	The Breast Cancer and Environment Research Program	Urine of the participants at the baseline (6–8 y)	GM of ∑DEHP metabolites (182 µg/g Cr [6 y], 152 µg/g Cr [7 y], and 152 µg/g Cr [8 y]) and LMWH (184 µg/g Cr [6 y], 136 µg/g Cr [7 y], and 163 µg/g Cr [8 y])	3 times until the last visit when girls were on average 14 y old (11–16 y)	BMI
9	Heggeseth^31^	2019	Cohort Study	U.S	335	CHAMACOS cohort study	Prenatal maternal urine	Median of MECPP, MEHHP, MEOHP, MnBP, and MiBP (24.05 µg/L, 14.8 µg/L, 10.75 µg/L, 20.7 µg/L, and 2.8 µg/L, respectively)	11 follow-up visits between ages 2 and 14 y	BMI
10	Hou^32^	2015	Cross-sectional study	Taiwan	308	270 normal adolescents (6.5–15 y) and 38 complainants (6.5–8.5 y)	Urine of the participants	GM of ∑DEHP, MnBP and MiBP (193.73 µg/L, 75.42 µg/L, and 47.06 µg/L, respectively)	When assessing phthalate exposure (6.5–8.5 y)	Obese (BMI), waist-to-hip ratio, Subcutaneous fat thickness
11	Kim^33^	2016	Cohort Study	South Korea	128	128 healthy pregnant women and their infants in 2012	Umbilical cord blood, newborns’ first urine	GM of MEHHP in maternal blood, maternal urine, cord blood, placenta, and newborns’ urine (0.31 µg/L, 18.23 µg/L, 0.,33 µg/L, 0.10 µg/L, and 5.83 µg/L, respectively), GM of MEOHP in maternal urine and newborns’ urine (15.88 µg/L, and 3.02 µg/L, respectively)	Perinatal	BMI z-score change during 3 months (Evaluation criterion for relative body mass increase was BMI z-score change over the 50^th^ percentile)
12	Kim^34^	2018	Cross-sectional study	South Korea	137	65 overweight children (6–13 y) and 72 controls	Urine of the participants	GM OF MECPP, MEOHP, and MEHHP (87.3 µg/g Cr, 29.5 µg/g Cr, and 36.8 µg/g Cr, respectively)	When assessing phthalates exposure (6–13 y)	BMI percentile
13	Lee^35^	2020	Cohort study	South Korea	481	EDC cohort	Prenatal maternal urine and urine of the participants	GM of molar ∑DEHP in prenatal maternal urine and children’s urine at 6 years of age (0.11 µM/L, and 0.33 µM/L, respectively) GM of ∑MnBP in prenatal maternal urine and children’s urine at 6 years of age (39.68 µg/L, and 70.00 µg/L, respectively)	6 y	BMI z-score, percentage of fat mass, fat mass index, percentage of skeletal muscle mass, skeletal muscle index
14	Maresca^36^	2016	Cohort study	U.S	424	CCCEH cohort	Prenatal maternal urine	GM of molar ∑DEHP metabolites, MiBP, and MnBP (0.29 µM/L, 8.81 µg/L, 37.58 µg/L)	5 y and 7 y	BMI z-score at 5 y and 7 y, percent of fat mass at 7 y, FMI at 7 y, WC at 7 y
15	Harley^37^	2017	Cohort study	U.S	219	CHAMACOS cohort study	Prenatal maternal urine, two times	GM of ∑DEHP, MECPP, MEHHP, MEOHP, MnBP, and MiBP in each measurement (0.2 and 0.2 nmol/mL, 25.9 and 32.4 µg/L, 15.1 and 18.8 µg/L, 11.2 and 13.8 µg/L, 22.8 and 28.5 µg/L, and 2.7 and 3.4 µg/L, respectively)	12 y	BMI z-score, WC
16	Saengkaew^38^	2017	Cross-sectional study	Thailand	155	Children aged 7–18 y	Urine of the participants	Median of MBP (216.47 µg/g Cr), detection rate 82.58%	When assessing phthalate exposure	BMI z-score, WC
17	Shoaff^39^	2017	Cohort study	U.S	219	HOME study	up to two times prenatally and six times from 1 to 8 y	GM of ∑DEHP, MiBP, and MnBP for children (86 µg/L, 4.8 µg/L, and 25 µg/L, respectively)	8 y	BMI z-score, WC, body fat percent
18	Smerieri^40^	2015	Cross-sectional study	Italy	72	41 obese children and 31 controls (mean age 12 y)	Urine of the participants	Detection rates of MECPP, MEOHP, and MEHHP were 80.5%, 87.8%, and 80.5% among obese group, and 38.7%, 74.2%, and 8.39% among control group, respectively	When assessing phthalate exposure	WC
19	Trasande^13^	2013	Cross-sectional study	U.S	2,884	NHANES 2003–2008 (children 6–19 y)	Urine of the participants	GM of ∑DEHP metabolite (0.358 µM/L among male and 0.360 among female) and ∑LMW metabolite (0.593 µM/L among male and 0.680 µM/L among female)	When assessing phthalate exposure	BMI z-score, overweight (BMI z-score > = 85^th^ percentile), and obesity (BMI z-score > = 95^th^ percentile)
20	Tsai^41^	2016	Cohort study	Taiwan	88	RAPIT program (6.0–10.5 y)	Estimated the total daily intake of DEHP, and urine of the participants	Mean of ∑DEHP metabolite 106.19 µg/g Cr	When participants were examined	Weight percentile and height percentile above 50^th^ percentile (based on the standards provided by the Ministry of Health and Welfare)
21	Vafeiadi^42^	2018	Cohort Study	Greece	500	Rhea Study	Prenatal maternal urine and urine of the participants	GM of molar ∑DEHP, MiBP and MnBP in prenatal maternal urine (0.1 µM/g Cr, 33.5 µg/g Cr, and 37.1 µg/g Cr, respectively) GM of molar ∑DEHP, MiBP, and MnBP in children’s urine (0.3 µM/gCr, 41.1 µg/g Cr, and 21.7 µg/g Cr, respectively)	4–6 y	BMI z-score
22	Valvi^43^	2015	Cohort study	Spain	391	INMA Spanish birth cohort	Prenatal maternal urine at 1^st^ and 3^rd^ trimester	GM of ∑DEHP metabolites, MnBP and MiBP (99.6 µg/gCr, 32.7 µg/gCr, and 33.0 µg/gCr, respectively)	Birth to 6 mos., 1, 4, and 7 y of age	BMI z-score, weight gain z-score (0–6 months)
23	Vrijheid^44^	2020	Cohort study	Europe	1,031	HELIX study (BiB in UK, EDEN in France, INMA in Spain, KANC in Lithuanina, MoBa and Rhea in Greece)	77 prenatal exposure and 96 childhood exposure including air pollutants, built environments, and biomarkers of chemical pollutants	Not described	BMI z-score (age-and-sex standardized z-scores)	BMI z-score
24	Wu^45^	2020	Cross-sectional study	U.S	2372	NHANES 2005–2010 (6–19 y)	Urine of the participants	GM of MiBP, 9.98 µg/L	When assessing phthalate exposure	BMI z-score
25	Xia^46^	2018	Cross-sectional study	China	159	PTHEC study, 69 overweight/obese children and 80 normal weight children	Urine of the participants	Median of MEOHP, MEHHP, and MnBP among normal participants (2.97 µg/L, 7.57 µg/L, and 13.68 µg/L, respectively) and among overweight/obese participants (2.6 µg/L, 6.5 µg/L, and 18.68 µg/L, respectively)	When assessing phthalate exposure	Overweight/obese
26	Xie^47^	2015	Case–control study	China	167	57 boys with constitutional delay for growth and puberty and 110 controls (11 y)	Urine of the participants	Median of ∑DEHP metabolites and MnBP among cases (20.06 µg/L and 37.43 µg/L, respectively), and among controls (12.85 µg/L and 15.56 µg/L, respectively)	When assessing phthalate exposure	Constitutional delay of growth and puberty
27	Zettergren^48^	2021	Cohort study	Sweden	100	BAMSE birth cohort	Urine of the participants at 4 years of age	GM of ∑DEHP metabolites and MnBP (331 µg/L, 296 µg/L)	24 y	BMI, WC, Body fat %, trunk fat % (Bio-electrical impedance analysis)
28	Zhang^11^	2014	Cross-sectional study	China	497	PTHEC study (8–13 y)	Urine of the participants	GM of ∑DEHP metabolites and MnBP; boys (8–10 y), 29.6 µg/L; boys (11–13 y), 21.9 µg/L; girls (8–10 y), 32.5 µg/L; girls (11–13 y), 16.5 µg/L	When assessing phthalate exposure	BMI z-score, body fat % (Yao’s formula)
29	Amin^49^	2017	Cross-sectional study	Iran	242		Urine of the participants	Mean of MEOHP, MEHHP, MEHP, MBzP, MBP, and MMP were 257.98 µg/L, 149.44 µg/L, 104.46 µg/L, 233.01 µg/L, 218.17 µg/L, and 59.82 µg/L	6–18 y	BMI z-score, WC
30	Ashley-Martin^50^	2021	Cross-sectional study	Canada	200	MIREC study	Urine of the participants	Twenty-two metabolites were measured. Median of ∑DEHP and ∑DiBP were 155 nmol/mL and 100 nmol/mL, respectively	2–5 y	BMI z-score
31	Ding^51^	2021	Cross-sectional study	China	463		Urine of the participants	Median of MECPP, MCMHP, MEOHP, MEHHP, MEHP, and ∑DEHP were 13.00 μg/L, 7.68 μg/L, 6.04 μg/L, 4.78 μg/L, 3.18 μg/L, and 34.56 μg/L	16–19 y	BMI, WHR, WHtR
32	Berger^52^	2021	Cohort study	U.S	309	CHAMACOS cohort study	Prenatal maternal urine	GM of ∑DEHP (0.2 nmol/mL)	5 y	BMI z-score
33	Li^53^	2021	Cohort study	China	814		Maternal urine in the 1st, 2nd and 3rd trimester of pregnancy	Median of ∑DEHP at 1st, 2nd, and 3rd trimesters were 0.09 nmol/mL, 0.06 nmol/mL, and 0.07 nmol/mL	average BMI z-score of 6-, 12- and 24-month	BMI z-score
34	Nidens^54^	2021	Cohort study	Germany	130		Prenatal maternal urine	GM of ∑HMWP (31.31 μg/gCr)	2 y	Weight gain (%) first 2 years of life
35	On^55^	2021	Cross-sectional study	South Korea	240		Urine of the participants	GM of MECPP, MEOHP, MEHHP, and MEHP (104.73 µg/g Cr, 33.96 µg/g Cr, and 14.54 µg/g Cr, respectively)	5–16 y	BMI percentile, weight percentile, and height percentile
36	Silva^56^	2021	Panel study	Netherland	471		Urine of the participants	Median of MECCP, MEOHP, and MEHHP were 0.94 nmol/L, 0.14 nmol/L, and 0.27 nmol/L	6y and 10 y	BMI z-score, Fat mass index
37	Hatch^57^	2008	Cross-sectional study	U.S	1,009 (6–19 y)	NHANES 1999–2002	Urine of the participants	GM of MEHHP among boys in 6–11 y and 12–19y were 39.6 μg/gCr and 21.1 μg/gCr, respectively GM of MEHHP among girls in 6–11 y and 12–19y were 39.1 μg/gCr and 18.2 μg/gCr, respectively	6–19y	BMI, WC
38	Wang^58^	2013	Cross-sectional study	China	259		Urine of the participants	GM of ∑DEHP (117.3 nmol/mL)	8–15y	BMI, WC
39	Bowman^59^	2019	Cohort study	Mexico	229	ELEMENT study	Urine of the participants	(boys) GM of ∑DEHP at 1st, 2nd, and 3rd trimester in prenatal maternal urine were 65.07 μg/L and 63.42 μg/L, and 78.60 μg/L (girls) GM of ∑DEHP at 1st, 2nd, and 3rd trimester in prenatal maternal urine were 71.03 μg/L and 75.97 μg/L, and 76.69 μg/L	8–14y (Visit 1) and 9–17y (Visit 2)	BMI z-score, WC, skinfold thickness

*MEHHP* mono-(2-ethyl-5-hydroxy-hexyl) phthalate; *MEOHP* mono-(2-ethyl-5-oxo-hexyl) phthalate; *MnBP* mono-n-butyl phthalate (MnBP); *MECCP* Mono-2-ethyl-5-carboxypentyl phthalate; *MBzP* Monobenzyl phthalatete; *BMI* body mass index; *WC* waist circumference.

### Prenatal exposure to phthalates and BMI z-scores

Supplementary Table S5 describes the studies investigating the association between prenatal exposure to phthalates and BMI. Among the 39 studies, 17 studies investigated the association between prenatal exposure to phthalates and BMI. The statistical significance of associations between phthalate metabolites and BMI of children is summarized in Supplementary Table S6.

**Figure 2:**
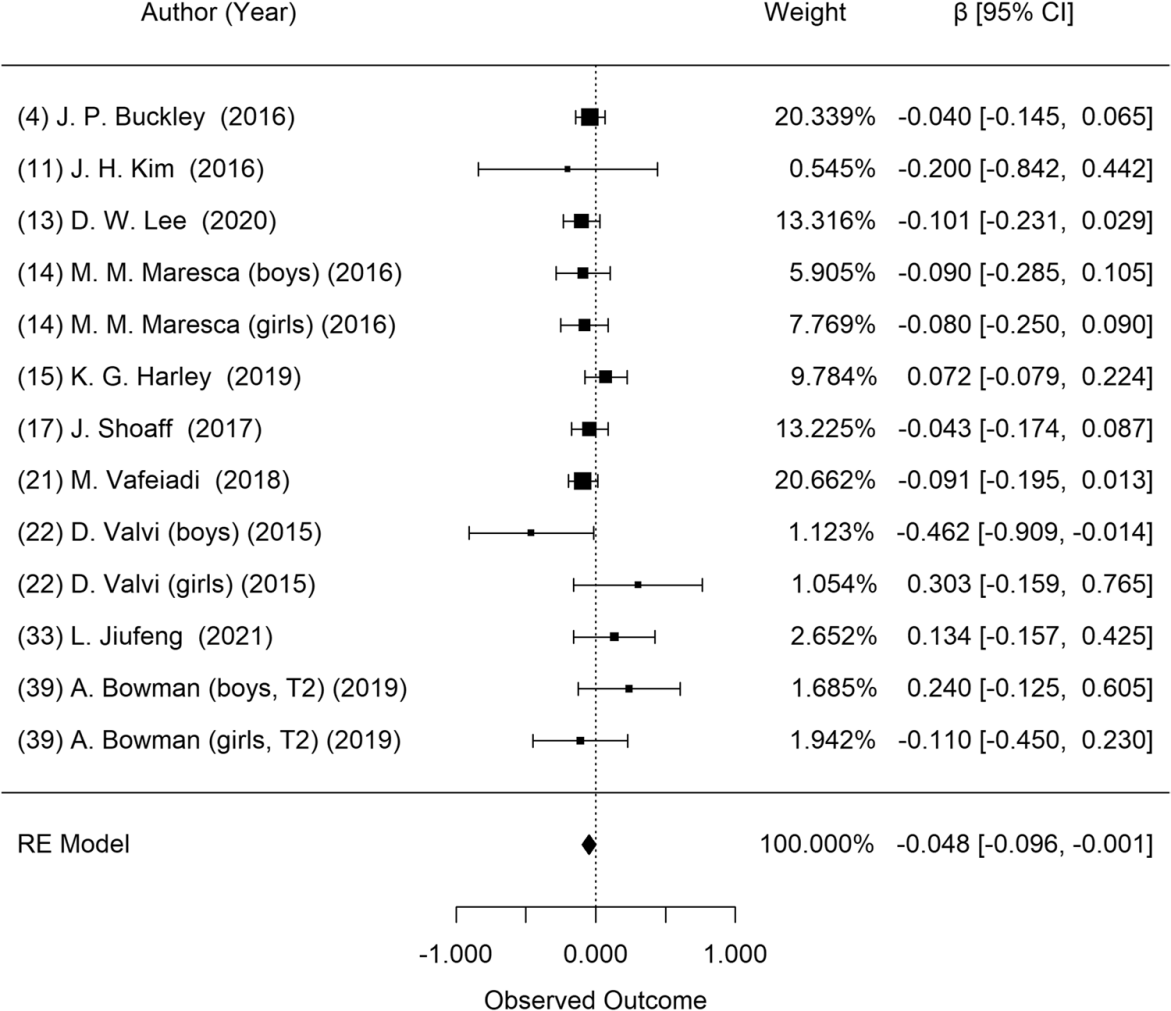
Forest plot of studies on the association of DEHP exposure with BMI z-scores: longitudinal studies. Estimates were standardized as β and 95% confidence intervals as one unit increase of natural log of DEHP metabolites.

Figure 2 shows the results of a meta-analysis on the association between prenatal DEHP exposure and BMI z-score in children. Ten studies presented eligible results for the meta-analysis. Data from Agay-Shay et al. were not included because they were derived from the same study population (INMA cohort) as that from Shoaff et al. K. Data from Berger et al. were not included because they presented an unadjusted beta coefficient from the Bayesian hierarchical model, and they were derived from the same study population (CHAMACOS cohort) with Harley et al. Heterogeneity among these studies was also not significant (*P* = 0.338). In the random effect model, there was a significant negative association between prenatal DEHP exposure and BMI z-score index (β = − 0.05; 95% CI: − 0.10, − 0.001). Visual inspection of the funnel plot revealed no asymmetry (Supplementary Fig. S1), and the Egger test showed no publication bias (*P* = 0.542).

**Figure 3:**
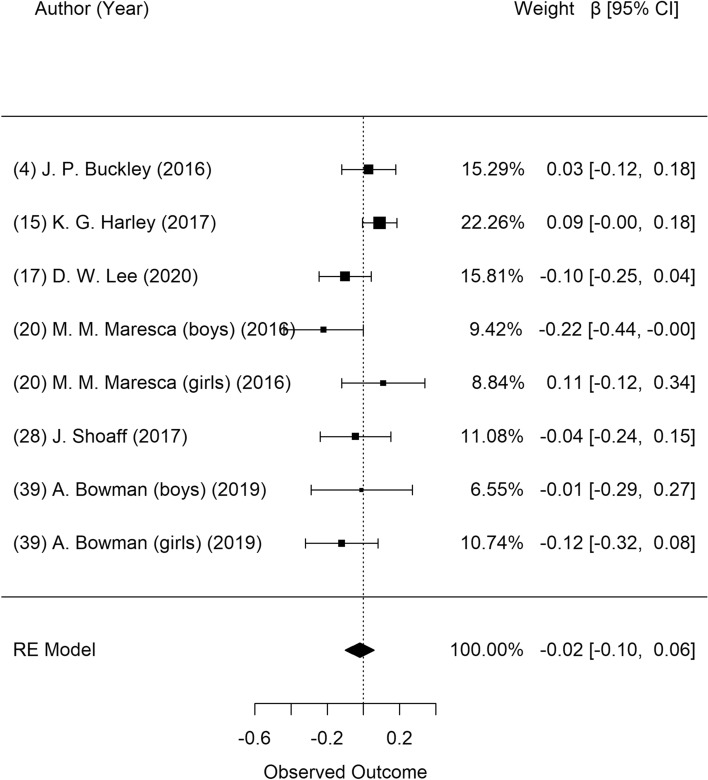
Forest plot of studies on the association of DBP exposure with BMI z-scores: longitudinal studies. Estimates were standardized as β and 95% confidence intervals as one unit increase of natural log of DBP metabolites.

Figure 3 shows the meta-analysis results on the association between prenatal DBP exposure and BMI z-scores in children. Seven studies presented data on BMI z-scores, and these were selected for the meta-analysis. Heterogeneity among these studies was suspected, but it was not statistically significant (*P* = 0.161). In the random-effects model, there was no significant association between prenatal DBP exposure and BMI z-score (β = − 0.02; 95% CI: − 0.10, 0.06). Visual inspection of the funnel plot revealed no asymmetry (Supplementary Fig. S2), and the Egger test showed no publication bias (*P* = 0.271).

### Prenatal exposure to phthalates and body fat percentage

Supplementary Table S7 describes studies that investigated the association between prenatal exposure to phthalates and body fat percentage. Among the 39 studies, seven were included. The results for this association were inconsistent, and only a limited number of studies reported statistical significance. The statistical significance of associations between phthalate metabolites and children's body fat percentage is summarized in Supplementary Table S8.

**Figure 4:**
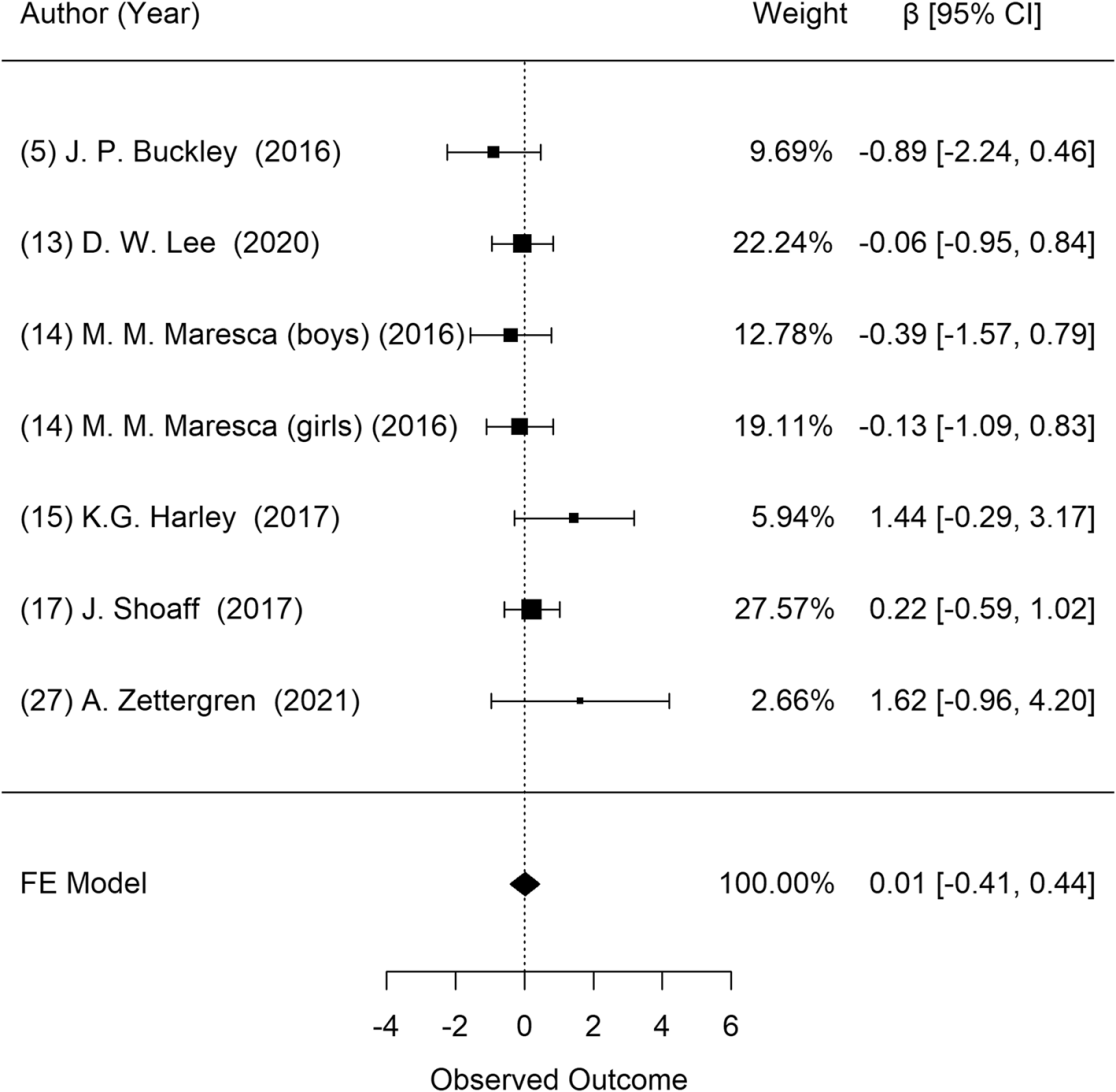
Forest plot of studies on the association of DEHP exposure with body fat percentage: longitudinal studies. Estimates were standardized as β and 95% confidence intervals as one unit increase of natural log of DEHP metabolites.

Figure 4 shows the results of the meta-analysis for the association between prenatal DEHP exposure and body fat percentage. Six studies presented body fat percentage data, which were chosen for the meta-analysis. Heterogeneity among these studies was not found (*P* = 0.358). In the random-effect model, no significant associations between prenatal DEHP exposure and body fat percentage were found (β = 0.01; 95% CI: − 0.41, 0.44). In addition, visual inspection of the funnel plot revealed no asymmetry (Supplementary Fig. S3), and the Egger test showed no publication bias (*P* = 0.287).

**Figure 5:**
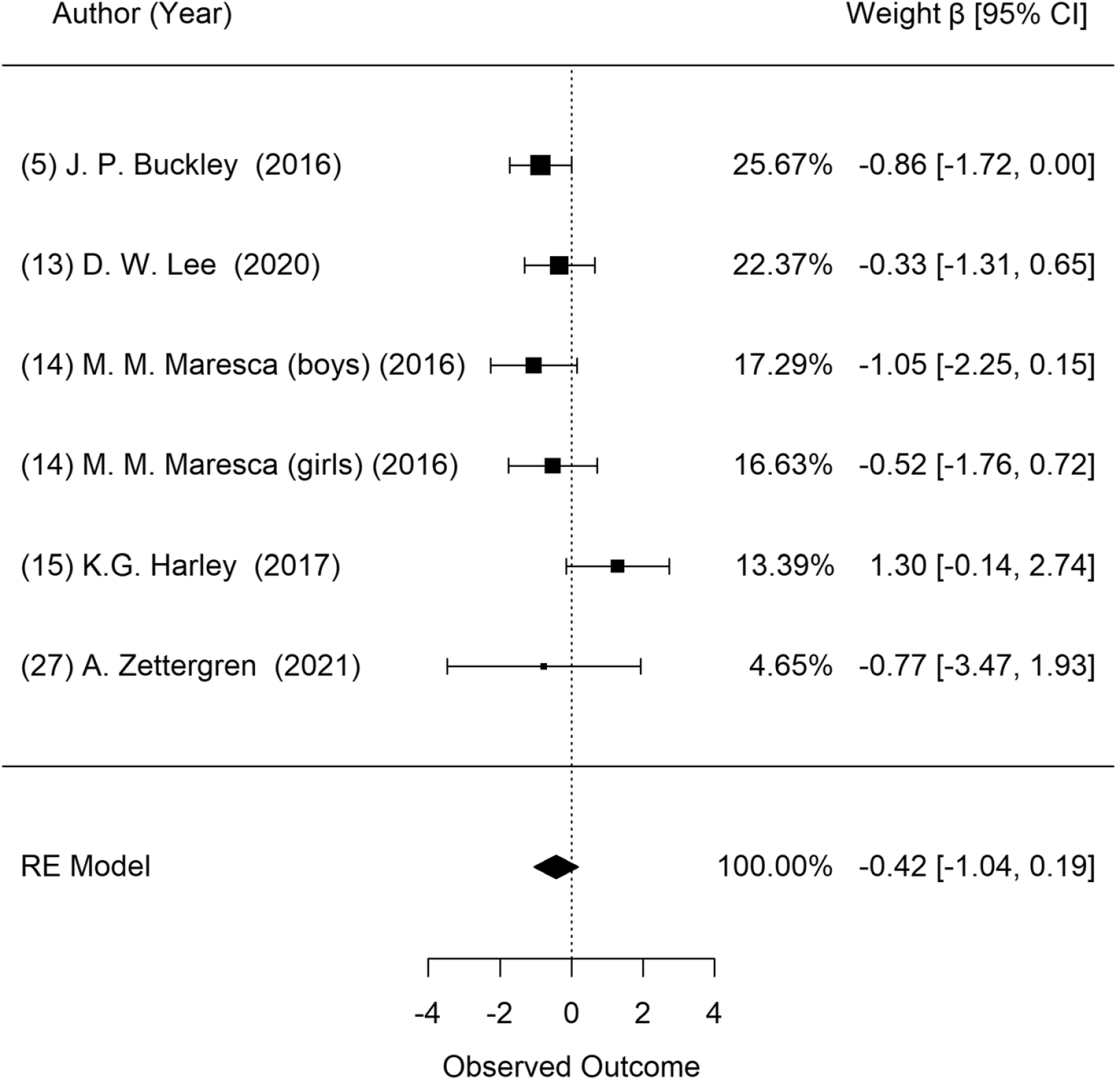
Forest plot of studies on the association of DBP exposure with body fat percentage: longitudinal studies. Estimates were standardized as β and 95% confidence intervals as one unit increase of natural log of DEHP metabolites.

Figure 5 shows the results of a meta-analysis on the association between prenatal DBP exposure and body fat percentage. Five studies presented data regarding body fat percentage, and these were selected for the meta-analysis. Heterogeneity among these studies was not found (*P* = 0.184), and there were no significant associations between prenatal DBP exposure and body fat percentage (β = − 0.42; 95% CI: − 1.04, 0.19). Furthermore, visual inspection of the funnel plot revealed no asymmetry (Supplementary Fig. S4), and the Egger test showed no publication bias (*P* = 0.601).

### Prenatal exposure to phthalates and other body composition indices

Supplementary Table S9 describes studies evaluating the association between the prenatal exposure to phthalates and body composition indices other than BMI or body fat percentage. Berman et al. assessed height, BMI, and body composition as measured by dual-energy X-ray absorptiometry (total fat percentage, total fat mass, and total lean mass), and the reported MiNP and MEHP were associated with decreased total lean mass^25^. Meanwhile, Buckley et al. used overweight/obesity defined by BMI z-score as the outcome variable^27^. Lee et al. also reported the association between phthalate metabolites and BMI z-score, fat mass percentage, fat mass index (FMI), skeletal muscle mass percentage, and skeletal muscle index (SMI) and reported that high levels of prenatal exposure to phthalates were significantly associated with decreased SMI among girls^35^. Maresca et al. reported that prenatal non-DEHP phthalate exposure was associated with lower BMI z-score, WC, and fat mass in boys during early childhood, contrary to their hypothesis^36^. Valvi et al. reported that weight gain Z-score was significantly associated with prenatal exposure to DEHP among boys^43^. Nidens et al. investigated the association between phthalate metabolites in prenatal maternal urine and weight gain (%) first 2 years of life, but it was not significant^54^.

### Postnatal exposure to phthalates and body composition indices

Supplementary Table S10 summarizes the studies assessing the association between the postnatal exposure to phthalates and the BMI. The results of the included studies were inconsistent, and there were limited studies that reported the association of BMI with phthalate metabolites as continuous variables. Chang et al. studied 152 children in Taiwan and reported non-significant associations of BMI with DEHP metabolites, MnBP, and MiBP^29^. Shaoff et al. also analyzed the data of 219 children from the HOME study and reported associations between BMI z-score at 8 years and DEHP metabolites at prenatal, 1, 2, 3, 4, 5, and 8 years of age were not statistically significant^39^. The only significant association was between a ten-fold increase in DEHP metabolites at 5 years of age and a 0.04-unit increase in BMI z-score. Trasande et al. reported that a unit increase in the natural log-transformed sum of LMWP was associated with a 0.07-unit increase in BMI z-score using the data of children surveyed at National Health and Nutrition Examination Survey (NHANES) 2003–2008^13^. Zettergren et al. investigated the participants’ phthalate metabolites at 4 years of age and their BMIs at 24 years of age and found that DiNP was associated with BMI, but DEHP and DBP were not^48^. A recent study in South Korea reported a significant association between urinary MEOHP and BMI percentile among children aged 5–16 years^55^, and Wang et al. also significant relationship between urinary sum of DEHP metabolites and BMI and WC. The statistical significance of associations between phthalate metabolites and BMI (and/or obesity) in children was summarized in Supplementary Table S11.

Supplementary Table S12 shows the studies on the association between the postnatal exposure to phthalates and the BMI. Chang et al. cross-sectionally studied 132 children and reported no association between phthalate metabolites and body fat percentage^29^, and Hou et al. studied 308 Taiwanese children and reported a significant association between the MnBP and MiBP and the waist-to-hip ratio^32^. Shaoff et al. analyzed the data of 219 children from the HOME study and showed significant associations between the waist circumference at 8 years of age and the sum of DEHP metabolites at 5 years of age^39^. There were significant associations between the body fat percentage at 8 years of age and the sum of DEHP metabolites at 1 and 5 years of age. In China, a case–control study on 57 boys with constitutional delay of growth and puberty and 110 controls reported that higher urinary phthalate metabolites were associated with constitutional delay of growth and puberty^47^. Another cohort study with 100 children reported non-significant associations between DEHP and DBP metabolites at 4 years of age and body indices until 24, including waist circumference, body fat percentage, and trunk fat percentage)^48^. Zhang et al. performed a cross-sectional study with 497 children in China and reported significant associations between phthalate exposure and fat distribution^60^. Ding et al. reported the significant association between waist-to-hip ratio and the sum of DEHP metabolites among children aged 16–19 years^51^.

## Discussion

### Main findings of the study

The systematic review and meta-analysis were performed to investigate the association between phthalates and physical growth in children. In the systematic literature review, a significant and negative association was found between the prenatal exposure to DEHP and the BMI z-score of the offspring, but there was no significant association between the prenatal exposure to DEHP and DBP and the body fat mass percentage of the offspring. Additionally, previous studies on the association between phthalates exposure in childhood and obesity were inconsistent in the systematic review.

### Prenatal exposure to phthalates and growth disturbance

We found that prenatal phthalate exposure and decreased offspring’s BMI were significantly associated. It implies that phthalates could act as disrupting chemicals on normal development instead of obesogens. Previous researches have focused on obesity, and found inconsistent results. Among children aged 5–12 years in the U.S., prenatal exposures to DEHP and DBP were associated with increased obesity^37^. However, Vafeiadi et al. investigated five-hundred mother–child dyads, and found that prenatal phthalate exposure was not significantly associated with overweight at ages 4–6 years^42^. Buckley et al. studied 707 children in the U.S. and found that BMI z-scores in girls aged 4–7 years were negatively associated with prenatal exposure to DEHP^27^. These inconsistent results lead to the idea that phthalates could not be obesogen. Our recent study suggested the selective association of phthalate exposure with the development of muscle mass than fat mass could explain the inconsistent associations between prenatal exposure to phthalates and BMI in children^35^. A cross-sectional study in the U.S. also showed that an increased urinary concentration of phthalate metabolites is associated with decreased lean mass^61^. If phthalate exposure could disturb the growth of muscle mass rather than induce obesity, it could explain the inconsistencies reported in previous studies regarding the association between prenatal exposure to phthalates and BMI during childhood.

### Possible mechanism

In the meta-analysis, prenatal exposure to phthalates was significantly associated with decreased BMI z-score but not with FMI. A possible explanation for this association is the antiandrogenic effects of phthalates on muscle development^5,62^. A murine study reported that androgen withdrawal mice showed decreased myofibrillar protein synthesis, and anabolic steroid administration reversed the effect^63^. Another study using mice also reported that testosterone had positive effects on muscle mass and the ultrastructure of muscles^64^. In an animal study, prenatal DEHP exposure led to decreased testosterone production in the offspring both in the fetal and postnatal period^65^. Several epidemiologic studies support that androgen is associated with muscle growth. A study with 50 boys and 50 girls aged 8–17 years reported that muscle strength was positively associated with testosterone levels^66^. Another study reported that testosterone is related with muscle mass and strength with a dose–response manner among hysterectomized women^67^. Furthermore, prenatal phthalate exposure is associated with decreased anogenital distance, which is positively related with antiandrogenic properties^68,69^. Increased phthalate metabolites were associated with decreased levels of serum testosterone in another human study^70^. Among children, the positive association between serum testosterone and SMI has been investigated^71^. Therefore, the antiandrogenic properties of phthalates could be an important link between prenatal exposure to phthalates and decreased SMI.

Inflammation is a possible mediator of disruption of muscle development following phthalate exposure. Phthalates exacerbate inflammatory response by increasing inflammatory cytokines^72^. A human study reported that DEHP exposure could induce interleukin-1β production in neonatal neutrophils^73^. In vitro study also reported that increased gene expression of inflammatory cytokines could be induced by DEHP^74^. Inflammatory cytokines are also associated with the inhibition of expression of myogenic miRNA in myoblasts and promoting muscle protein degradation^75,76^. Therefore, it could be inferred that inflammation due to phthalates could be associated with decreased SMIs.

Insulin-like growth factor-1 (IGF-1) could be another possible link of the association of phthalates with muscle mass. IGF-1 pathway is an important regulator of muscle growth processes in children^77^. Several epidemiologic studies have reported that urinary phthalate metabolites are negatively associated with IGF-1^41,78–80^. These studies support that phthalates could lead to decreased muscle growth in children via IGF-1.

### Phthalates exposure in children and body composition indices

The results of searched studies in the systematic review were inconsistent for the associations between the phthalates exposure in children and their body composition. Several researchers reported that phthalate exposure in children could be related with obesity, although obesity was inconsistently associated with phthalate metabolites, and the number of studies was limited to perform the meta-analysis. As one of the results with a significant association, a cross-sectional study involving 845 Danish children aged 4–9 years reported that children’s height and weight are negatively associated with urinary phthalate metabolites^79^. However, several studies showed a positive association between phthalates and obesity. A study that used NHANES data reported that LMWP could be associated with increased BMI z-score^13^, and a longitudinal study in the U.S. also noted that obesity at 8 years of age was associated with phthalate exposure at 5 years of age^39^. These studies suggested that the role of peroxisome proliferator activated receptors (PPARs), nuclear hormone receptors that have regulatory roles in adipogenesis and lipid storage, is important to induce adipogenesis and obesity^81–83^. Because phthalate exposure is associated with decreased thyroid hormone^84^, hormonal homeostasis can be disturbed due to phthalates, leading to fat accumulation and obesity. A Chinese metabolome study investigated 69 overweight/obese children and 80 normal-weight children. It was reported that urinary MnBP concentration differed between the two groups and was associated with arginine, proline, and butyraldehyde^46^. However, several studies had no significant associations between phthalates, obesity, and BMI^13,28,29,38,39,44^.

Some researchers argued that the association between urinary phthalates metabolites and obesity was not derived from the causal association between phthalates exposure in children and obesity. For instance, the recent study that explained the mechanism for cross-sectional studies for the association between phthalates and higher BMI demonstrated that the higher energy intake in the overweight and obese could result in the concomitant higher phthalates exposure^85^. Additionally, ultra-processed food consumption is associated with overweight and weight gain^86^, and is also associated with urinary phthalates metabolites^87^. Therefore, cross-sectionally observed association between phthalates metabolites and obesity might reflect the association of the dietary pattern and the amount of consumption with obesity. Additionally, urinary phthalate metabolite may be measured higher among children with more adipose and muscle mass. Given the absorption, distribution, metabolism, and excretion of phthalates, absorbed phthalates in the human body distribute mainly in the intestine and liver, and they are rapidly excreted.

On the other hand, a relatively small portion of absorbed phthalates is distributed in fat and muscle tissue. Still, they are excreted slower than those in the intestine and liver, resulting in a relatively higher proportion of phthalates in the human body^88^. Therefore, observed cross-sectional associations between phthalates and obesity in children might not be causal. Inconsistent results and related factors make it difficult to conclude the association between phthalates exposure in childhood and weight gain. Studies with longitudinal design and studies suggesting plausible mechanism, such as hormonal, epigenetic and/or metabolomic changes, are needed in the future.

### Exposure assessment for phthalates

It has been assumed that a single measure of phthalate metabolites can adequately reflect exposure across the studies. All studies included in the meta-analysis also had the same assumption. Assessing DEHP exposures may be inconclusive because various metabolites of DEHP are rapidly metabolized in vivo and quickly excreted. As the excretion half-lives of DEHP metabolites are 0.5–3.0 days^20^, urine biomarkers can only reflect recent exposure. However, all studies included in the meta-analysis considered DEHP metabolites in the mothers’ or children’s urine. In all longitudinal studies, DEHP metabolites were assessed at a one-time point, rather than repeated measurements in a few-day interval. Included cross-sectional studies were also measured DEHP metabolites only once from children’s urine.

The temporal stability of DEHP metabolites over weeks to months has been studied. The daily variation of phthalates' urinary metabolites was investigated using urine samples of fifty participants on eight consecutive days, and reported intra-class coefficients of urinary DEHP metabolites as 0.20–0.34^89^. Another study reported one spot urine sample could predict the three-month average concentration of DEHP metabolites with sensitivity and specificity as 0.56 and 0.83, respectively^90^. It has been suggested that DEHP metabolites measured in the spot urine showed reasonable temporal stability for weeks to months, although it has limitations on stability^91–95^. In addition, a recent study investigated 805 urine samples of 16 volunteers for 6 months and suggested that adequately classifying the exposure level of participants requires several samples per subject^96^. In this systematic review, no studies measured phthalates repeatedly in a short time period to measure phthalates exposure more accurately. Therefore, all studies included in our meta-analyses assumed implicitly or explicitly that a single measurement could reflect exposure over a considerable period.

### Strengths and limitations

To overcome the inconclusive results on the association between phthalate exposure and children’s growth^17^, we rationally and preferentially selected the estimate for the association between phthalates (exposure) and body composition indices (outcome). We used the sum of phthalate metabolites to assess the total exposure amount because the molar sum of several metabolites of DEHP is currently considered the best estimate of exposure rather than a simple mass sum of DEHP metabolites. Furthermore, the time points of measurement for phthalate exposure (prenatal or postnatal) and the methods for assessing body composition indices, including BMI, BMI z-score, and body fat percentage, differ across studies. In the present study, we attempted to collectively analyze the results in a meta-analysis with the abovementioned methods, which was also described in a previous meta-analysis study^97^. Therefore, we estimated the up-to-date summarized results for the association of prenatal phthalates exposure and body composition indices in children.

This study has several limitations. First, the calibration of the amount of exposure to phthalates considering the duration of exposure is not assessed in the systematic review and meta-analysis, because it is practically impossible. Second, the included studies had limited information and had methodological differences^98^, although standardized values from β estimates and 95% CIs were used to perform meta-analyses. If raw data can be obtained and pooled analysis is performed, more robust results may be expected. In the studies we reviewed, phthalate metabolites were measured from spot urine samples of participants. There is no study with repetitive measurement for accurate phthalates measurement for the association between phthalates and body composition indices. In the future, more repetitive methods such as using mean levels of various phthalate metabolites assessed at multiple time points could increase the precision and accuracy of predicting phthalate exposure^99^.

## Conclusion

This systematic review and meta-analysis showed that prenatal exposure to phthalates is significantly associated with low BMI in children, but not with body fat mass. In addition, prenatal phthalate exposure may affect the disturbance of normal growth of children rather than act as an obesogen. Future studies on the health effects of phthalates should consider their detrimental effects on the expected growth of children. Furthermore, it is necessary to administer stricter and broader regulations on phthalates in living environments.

## Supplementary Information

Supplementary Information.
