# Supplementary material for: Prenatal exposure to phthalate and decreased body mass index of children: a systematic review and meta-analysis
Source: Sci Rep. 2022 May 27;12:8961. doi: 10.1038/s41598-022-13154-9 (PMC9142490; doi:10.1038/s41598-022-13154-9)
Supplement: Supplementary file 1 — Supplementary Information. [file 41598_2022_13154_MOESM1_ESM.docx]

**Supplementary Material**

**Prenatal exposure to phthalate and decreased body mass index of children: a systematic review and meta-analysis**

Dong-Wook Lee^1^, Hyun-Mook Lim^2^, Joong-Yub Lee^2^, Kyung-Bok Min^2^, Choong-Ho Shin^3^, Young-Ah Lee^3^, and Yun-Chul Hong^4^

^1^Public Healthcare Center, Seoul National University Hospital, 101 Daehak-ro, Jongno-gu, Seoul 03080 Republic of Korea

^2^Department of Preventive Medicine, Seoul National University College of Medicine, 103 Daehak-ro, Jongno-gu, Seoul 03080 Republic of Korea

^3^Department of Pediatrics, Seoul National University College of Medicine, 103 Daehak-ro, Jongno-gu, Seoul 03080 Republic of Korea

^4^Department of Humans Systems Medicine, Seoul National University College of Medicine, 103 Daehak-ro, Jongno-gu, Seoul 03080 Republic of Korea

**Corresponding author:**

Yun-Chul Hong MD, PhD

Department of Humans Systems Medicine, College of Medicine, Seoul National University

103 Daehak-ro, Jongno-gu, Seoul 110-799, Republic of Korea

E-mail: ychong1@snu.ac.kr

Telephone: 822-740-8394

**Table of Contents**

Supplementary figure S1. Funnel plot of studies on the association of DEHP exposure with BMI z-scores: longitudinal studies １

Suppelemntary figure S2. Funnel plot of studies on the association of DBP exposure with BMI z-scores: longitudinal studies １

Supplementary Table S1. Comprehensive search strategies for PubMed and EMBASE ３

Supplementary Table S2. Reasons for exclusion in full-text review. ４

Supplementary Table S3. Newcastle-Ottawa Quality Assessment Scale for included longitudinal Studies ７

Supplementary Table S4. Newcastle-Ottawa Quality Assessment Scale for included cross-sectional studies ８

Supplementary Table S5. Description of papers accessing the association between prenatal exposure to phthalates and BMI ９

Supplementary Table S6. Statistical significance of associations between prenatal phthalates exposure and BMI in children １３

Supplementary Table S7. Description of studies on the association between prenatal exposure to phthalates and body fat percentage １４

Supplementary Table S8. Statistical significance of associations between prenatal phthalates exposure and percent fat mass in children １６

Supplementary Table S9. Description of studies on the association between prenatal exposure to phthalates and other body composition indices １７

Supplementary Table S10. Description of studies on the association between postnatal exposure to phthalates and BMI ２０

Supplementary Table S11. Statistical significance of associations of phthalates exposure at children with BMI, obese, and obesity in children ２４

Supplementary Table S12. Description of studies on the association between postnatal exposure to phthalates and body indices other than BMI ２５


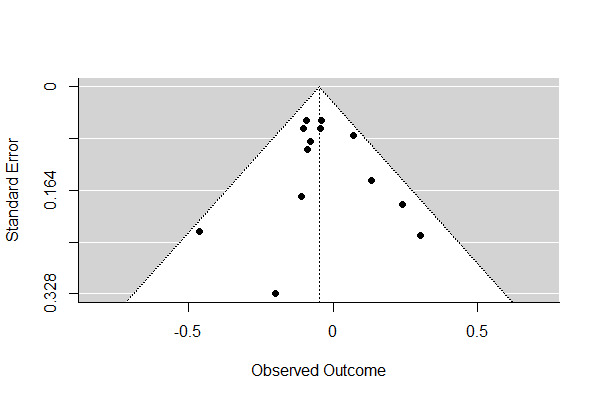


**Supplementary figure S1. Funnel plot of studies on the association of DEHP exposure with BMI z-scores: longitudinal studies**


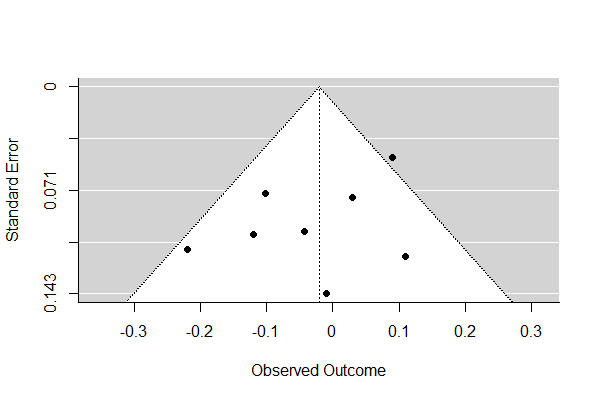


**Suppelemntary figure S2. Funnel plot of studies on the association of DBP exposure with BMI z-scores: longitudinal studies**


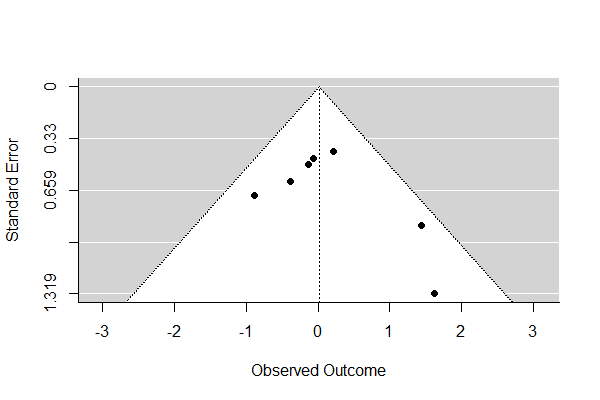


**Supplementary figure S3. Funnel plot of studies on the association of DEHP exposure with body fat percentage: longitudinal studies**


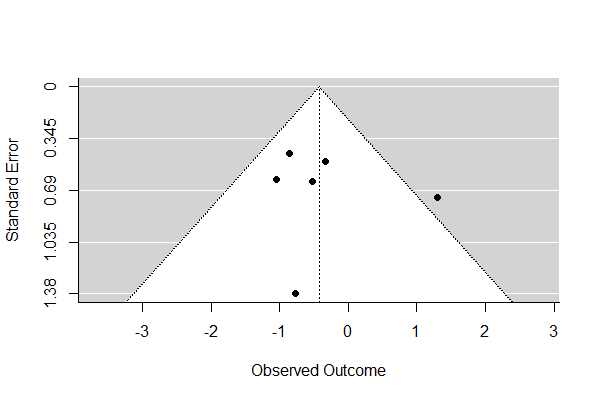


**Supplementary figure S4. Funnel plot of studies on the association of DEHP exposure with body fat percentage: longitudinal studies**

**Supplementary Table S1. Comprehensive search strategies for PubMed and EMBASE**

| **PubMed** |  |
| --- | --- |
| Component 1: exposure | "phthalate" [tw] OR "phthalates" [tw] OR “diethylhexyl phthalate” [MeSH] OR “diethylhexyl phthalate” [tiab] OR di-2-ethylhexylphthalate [tiab] OR DEHP [tiab] OR "dibutyl phthalate" [MeSH] OR "dibutyl phthalate"[tiab] OR DBP [tiab] |
| Component 2: health problem | Growth [MeSH] OR “Body Mass Index” [MeSH] OR BMI [tiab] OR obesity [MeSH] OR obesity [tiab] OR "body composition" [MeSH] OR “body fat distribution” [MeSH] OR adiposity [MeSH] OR adiposity [tiab] OR overweight [MeSH] OR overweight [tiab] OR “quetelet index” [tiab] OR “weight gain” [MeSH] OR “weight gain” [tiab] OR adipogenesis [MeSH] OR adipogenesis [tiab]ORr "fat mass index" [tiab] OR “body weight” [MeSH] OR ("body weight" [tiab] NOT "kg body weight" [tiab] NOT "body weight/day" [tiab]) OR obesogenic [tiab] OR "Muscle, Skeletal" [MeSH] OR "skeletal muscle index" [tiab] OR "body Height" [Mesh] OR "height" [tiab] |
| Component 3: humanstudies | NOT (animals [MeSH Terms] NOT humans [MeSH Terms]) |
| **EMBASE** |  |
| Component 1: exposure | "phthalate"/exp OR "phthalates"/exp OR “diethylhexyl phthalate”:ab,ti OR di-2-ethylhexylphthalate:ab,ti OR DEHP:ab,ti OR "dibutyl phthalate":ab,ti OR DBP:ab,ti |
| Component 2: health problem | Growth:ab,ti OR “Body Mass Index”:ab,ti OR BMI:ab,ti OR obesity:ab,ti OR obesity:ab,ti OR "Body Composition":ab,ti OR “body fat distribution”:ab,ti OR adiposity:ab,ti OR adiposity:ab,ti OR overweight:ab,ti OR overweight:ab,ti OR “quetelet index”:ab,ti OR “weight gain”:ab,ti OR “weight gain”:ab,ti OR adipogenesis:ab,ti OR adipogenesis:ab,ti OR "fat mass index":ab,ti OR “body weight”:ab,ti OR obesogenic:ab,ti OR "Muscle, Skeletal":ab,ti OR "skeletal muscle index":ab,ti OR "height":ab, ti |
| Component 3: human studies | NOT animals/ NOT humans.sh. |

**Supplementary Table S2. Reasons for exclusion in full-text review.**

| **ID** | **Firt author** | **Year** | **Title** | **Journal** | **Reason for exclusion** |
| --- | --- | --- | --- | --- | --- |
| 1 | M. Boas | 2010 | Childhood exposure to phthalates: associations with thyroid function, insulin-like growth factor I, and growth | Environ Health Perspect | Not able to use the size of the association |
| 2 | F. Brucker-Davis | 2010 | Exposure to selected endocrine disruptors and neonatal outcome of 86 healthy boys from Nice area (France) | Chemosphere | Not able to use the size of the association |
| 3 | J. L. Campbell | 2018 | Excretion of Di-2-ethylhexyl phthalate (DEHP) metabolites in urine is related to body mass index because of higher energy intake in the overweight and obese | Environ Int | Not able to use the size of the association Not relavent study for the association between DEHP and DBP levels and the physical growth of children |
| 4 | Y. H. Chiu | 2018 | Evaluating effects of prenatal exposure to phthalate mixtures on birth weight: A comparison of three statistical approaches | Environ Int | Not reported the association between DEHP and DBP levels and the physical growth of children |
| 5 | J. Choi | 2014 | Association between some endocrine-disrupting chemicals and childhood obesity in biological samples of young girls: a cross-sectional study | Environ Toxicol Pharmacol | Not reported the association between DEHP and DBP levels and the physical growth of children |
| 6 | M. de Cock | 2014 | First year growth in relation to prenatal exposure to endocrine disruptors - a Dutch prospective cohort study | Int J Environ Res Public Health | Presented outcomes in irrelevant forms (the methods of stiatiscal analyses were not described) |
| 7 | A. C. Dirtu | 2013 | Phthalate metabolites in obese individuals undergoing weight loss: Urinary levels and estimation of the phthalates daily intake | Environ Int | Not relavent study for the association between DEHP and DBP levels and the physical growth of children |
| 8 | E. E. Hatch | 2010 | Association of endocrine disruptors and obesity: perspectives from epidemiological studies | Int J Androl | Not relavent study for the association between DEHP and DBP levels and the physical growth of children |
| 9 | B. Kolena | 2017 | Occupational phthalate exposure and health outcomes among hairdressing apprentices | Hum Exp Toxicol | Not relavent study for the association between DEHP and DBP levels and the physical growth of children; no description for the age distribution of the study participants in the abstract |
| 10 | J. E. Lim | 2020 | Urinary bisphenol A, phthalate metabolites, and obesity: do gender and menopausal status matter? | Environ Sci Pollut Res Int | letter, commentary, or review articles |
| 11 | N. Milošević | 2020 | Could phthalates exposure contribute to the development of metabolic syndrome and liver disease in humans? | Environ Sci Pollut Res Int | Not relavent study for the association between DEHP and DBP levels and the physical growth of children; no description for the age distribution of the study participants in the abstract |
| 12 | N. Milošević | 2017 | Potential influence of the phthalates on normal liver function and cardiometabolic risk in males | Environ Monit Assess | Not relavent study for the association between DEHP and DBP levels and the physical growth of children; no description for the age distribution of the study participants in the abstract |
| 13 | W. Perng | 2020 | Exposure to Endocrine-Disrupting Chemicals During Pregnancy Is Associated with Weight Change Through 1 Year Postpartum Among Women in the Early-Life Exposure in Mexico to Environmental Toxicants Project | J Womens Health (Larchmt) | Not reported the association between DEHP and DBP levels and the physical growth of children |
| 14 | I. Petrovičová | 2016 | Occupational exposure to phthalates in relation to gender, consumer practices and body composition | Environ Sci Pollut Res Int | Not reported the association between DEHP and DBP levels and the physical growth of children |
| 15 | E. M. Philips | 2020 | Exposures to phthalates and bisphenols in pregnancy and postpartum weight gain in a population-based longitudinal birth cohort | Environ Int | Not reported the association between DEHP and DBP levels and the physical growth of children |
| 16 | Y. Rodríguez-Carmona | 2019 | Phthalate exposure during pregnancy and long-term weight gain in women | Environ Res | Not reported the association between DEHP and DBP levels and the physical growth of children |
| 17 | S. Sathyanarayana | 2016 | First Trimester Phthalate Exposure and Infant Birth Weight in the Infant Development and Environment Study | Int J Environ Res Public Health | Not reported the association between DEHP and DBP levels and the physical growth of children |
| 18 | K. Svensson | 2021 | Prenatal exposures to mixtures of endocrine disrupting chemicals and children's weight trajectory up to age 5.5 in the SELMA study | Scientific reports | Not relavent study for the association between DEHP and DBP levels and the physical growth of children |
| 19 | C. Y. Tang | 2012 | Influence of polluted SY River on child growth and sex hormones | Biomed Environ Sci | Not reported the association between DEHP and DBP levels and the physical growth of children |
| 20 | V. Tran | 2017 | Prenatal phthalate exposure and 8-isoprostane among Mexican-American children with high prevalence of obesity | J Dev Orig Health Dis | Not relavent study for the association between DEHP and DBP levels and the physical growth of children |
| 21 | T. P. van der Meer | 2021 | Endocrine disrupting chemicals during diet-induced weight loss - A post-hoc analysis of the LOWER study | Environ Res | Not relavent study for the association between DEHP and DBP levels and the physical growth of children |
| 22 | L. Yaghjyan | 2015 | Associations of urinary phthalates with body mass index, waist circumference and serum lipids among females: National Health and Nutrition Examination Survey 1999-2004 | Int J Obes (Lond) | Not relavent study for the association between DEHP and DBP levels and the physical growth of children |
| 23 | Y. Zhang | 2019 | Association between exposure to a mixture of phenols, pesticides, and phthalates and obesity: Comparison of three statistical models | Environ Int | Not relavent study for the association between DEHP and DBP levels and the physical growth of children |

**Supplementary Table S3. Newcastle-Ottawa Quality Assessment Scale for included longitudinal Studies**

| **ID** | **First author** | **Study Design** | **Newcastle-Ottawa Scale** | | | | | | | | |
| --- | --- | --- | --- | --- | --- | --- | --- | --- | --- | --- | --- |
|  |  |  | **S1** | **S2** | **S3** | **S4** | **C** | **O1** | **O2** | **O3** | **Sum** |
| 1 | K. Agay-Shay | Cohort study | * | * | * | * | ** | * | * | * | 9 |
| 2 | Y. E. Berman | Cohort study | * | * | * | * | ** | * | * | * | 9 |
| 3 | J. Botton | Cohort study | * | * | * | * | ** | * | * | * | 9 |
| 4 | J. P. Buckley | Cohort study | * | * | * | * | ** | * | * | * | 9 |
| 5 | J. P. Buckley | Cohort study | * | * | * | * | ** | * | * | * | 9 |
| 8 | A. L. Deierlein | Cohort study | * | * | * | * | * | * | * | * | 8 |
| 9 | B. C. Heggeseth | Cohort Study | * | * | * | * | ** | * | * | * | 9 |
| 11 | J. H. Kim | Cohort Study | * | * | * | * | ** | * | * | * | 9 |
| 13 | D. W. Lee | Cohort study | * | * | * | * | ** | * | * | * | 9 |
| 14 | M. M. Maresca | Cohort study | * | * | * | * | ** | * | * | * | 9 |
| 15 | K. G. Harley | Cohort study | * | * | * | * | ** | * | * | * | 9 |
| 17 | J. Shoaff | Cohort study | * | * | * | * | ** | * | * | * | 9 |
| 20 | Y. A. Tsai | Cohort study | * | * | * |  | ** | * | * | * | 8 |
| 21 | M. Vafeiadi | Cohort Study | * | * | * | * | ** | * | * | * | 9 |
| 22 | D. Valvi | Cohort study | * | * | * | * | ** | * | * | * | 9 |
| 23 | M. Vrijheid | Cohort study | * | * | * | * | ** | * | * | * | 9 |
| 27 | A. Zettergren | Cohort study | * | * | * | * | ** | * | * | * | 9 |
| 32 | K. Berger | Cohort study | * | * | * | * | ** | * | * | * | 9 |
| 33 | L. Jiufeng | Cohort study | * | * | * | * | ** | * | * | * | 9 |
| 34 | N. Nidens | Cohort study | * | * | * | * | ** | * | * | * | 9 |
| 36 | C. Silva | Panel study | * | * | * | * | ** | * | * | * | 9 |
| 39 | A. Bowman | Cohort study | * | * | * | * | ** | * | * | * | 9 |

**Supplementary Table S4. Newcastle-Ottawa Quality Assessment Scale for included cross-sectional studies**

| ID | First author | Study Design | Newcastle-Ottawa Scale | | | | | | | | |
| --- | --- | --- | --- | --- | --- | --- | --- | --- | --- | --- | --- |
|  |  |  | S1 | S2 | S3 | S4 | C | O1 | O2 | Sum | |
| 6 | M. C. Buser | Cross-sectional study | * |  |  | ** | ** | ** | * | 8 |  |
| 7 | C. H. Chang | Cross-sectional study | * |  |  | ** | ** | ** | * | 8 |  |
| 10 | J. W. Hou | Cross-sectional study |  |  | * | ** | ** | * | * | 7 |  |
| 12 | S. H. Kim | Cross-sectional study |  |  | * | ** | * | * | * | 6 |  |
| 16 | T. Saengkaew | Cross-sectional study |  |  | * | ** |  | ** |  | 5 |  |
| 18 | A. Smerieri | Cross-sectional study |  |  | * | ** |  | ** |  | 5 |  |
| 19 | L. Trasande | Cross-sectional study | * |  |  | ** | ** | ** | * | 8 |  |
| 24 | B. Wu | Cross-sectional study | * |  |  | ** | ** | ** | * | 8 |  |
| 25 | B. Xia | Cross-sectional study | * |  |  | ** |  | ** | * | 6 |  |
| 26 | C. Xie | Case-control study | * | * |  |  | ** | * |  | 5 | |
| 28 | Y. Zhang | Cross-sectional study | * |  |  | ** | ** | ** | * | 8 | |
| 29 | M. M. Amin | Cross-sectional study | * |  |  | ** | ** | ** | * | 8 | |
| 30 | J. Ashley-Martin | Cross-sectional study | * |  |  | ** | ** | ** | * | 8 | |
| 31 | S. Ding | Cross-sectional study | * |  |  | ** | ** | ** | * | 8 | |
| 35 | J. On | Cross-sectional study |  |  |  | ** | ** | ** | * | 7 | |
| 37 | E.E. Hatch | Cross-sectional study | * |  |  | ** | ** | ** | * | 8 | |
| 38 | H. Wang | Cross-sectional study |  |  |  | ** | ** | ** | * | 7 | |

**Supplementary Table S5. Description of papers accessing the association between prenatal exposure to phthalates and BMI**

| **ID** | **First author** | **Year** | **Exposure assessment** | **Timing of outcome assessment** | **Statistical analysis** | **Estimates Type** | **Estimates** | **Adjustment variables** |
| --- | --- | --- | --- | --- | --- | --- | --- | --- |
| 1 | K. Agay-Shay | 2015 | Maternal urine in the 1^st^ and 3^rd^ trimesters of pregnancy | 7 y | Multiple linear regression | β and 95% CI | (BMI z-scores) MECPP highest tertile (vs. lowest tertile), -0.26 (95% CI: -0.55, 0.04); MnBP highest tertile (vs. lowest tertile), -0.08, (95% CI: -0.37, 0.22) | sex, gestational age at birth, birth weight, age, maternal country of origin, maternal age at delivery, maternal pre-pregnancy BMI, maternal weight gain during pregnancy, maternal social class, breastfeeding duration, and maternal smoking during pregnancy |
| 2 | Y. E. Berman | 2020 | Maternal urine in the 2^nd^ and 3^rd^ trimesters of pregnancy | 1, 2, 3, 5, 8, 10, 14, 17, and 20 y | Linear mixed effect model | β and 95% CI or tertial and 95% CI | (BMI Z-score) Marginal mean z-score of 2^nd^ tertile for MiBP in 2–11 y [0.46 (95% CI: 0.28, 0.64)] and in 11–20 y [0.43 (95% CI: 0.24, 0.63)]. Marginal mean z-score of 2^nd^ tertile for ∑low molecular phthalates metabolites in 2–11 y [0.44 (95% CI: 0.26, 0.62)] | age, gestational age at birth, birthweight, and maternal pre-pregnancy BMI |
| 3 | J. Botton | 2016 | Maternal urine in the 2^nd^ trimester | 5 y | Multiple linear regression | β and 95% CI | (BMI) IQR increase of MEP 0.17 kg/m^2^ (0.04, 0.30); ∑DEHP metabolites and DBP metabolites in maternal urine were not significantly associated with BMI at 5 years of age (data was not shown) | recruitment center, maternal height, BMI using self-reported pre-pregnancy weight, smoking during pregnancy, education level, age, weight gain during pregnancy, and parity. |
| 4 | J. P. Buckley | 2016 | Prenatal maternal urine | 4–9 y | Linear mixed effect model | β and 95% CI or OR and 95% CI | **(BMI z-score) β for natural log ∑DEHP metabolites (-0.04 [95% CI: -0.15, 0.07]),  β for natural log MnBP (0.03 [95% CI: -0.12, 0.18])** (Overweight/obese) OR for standard deviation increase in natural log ∑DEHP metabolites, MnBP and MiBP (0.87 [0.53, 1.4], 1.0 [0.51, 2.0], and 0.84 [0.44, 1.6], respectively) | cohort, maternal race/ethnicity, maternal age at delivery, maternal education, maternal work status during pregnancy, maternal pre-pregnancy BMI, gestational weight gain, maternal smoking during pregnancy, calendar date of urine collection, and parity. |
| 8 | A. L. Deierlein | 2016 | Urine of the participants at the baseline (6–8 y) | 3 times until the last visit when girls were on average 14 y old (11–16 y) | Linear mixed effect model | β and 95% CI | (BMI) β of ∑DEHP high vs. low, 0.63 (-0.17, 1.4); LMWH high versus low, 1.2 (0.28, 2.1) | age, age^2^, race/ethnicity, age×phthalate categories, age^2^×phthalate categories, and race/ethnicity×age. |
| 9 | B. C. Heggeseth | 2019 | Prenatal maternal urine | 11 follow-up visits between ages of 2 and 14 y | GAM, Growth Mixture Models, FPCA, Regression Trees, Random Forest | β and 95% CI | (β for Principal component 1 for BMI trajectories, boy) MECPP 0.2 (-2.18, 2.58), MnBP 1.18 (-0.63, 3.00), and MiBP 0.04 (-1.57, 1.65); (β for Principal component 1 for BMI trajectories, girls) MECPP 1.46 (-0.63, 3.56), MnBP 0.57 (-1.38, 2.52), and MiBP 0.22 (-1.45, 1.90) | maternal pre-pregnancy BMI, gestational weight gain, diet quality index during pregnancy, smoking during pregnancy, education, marital status, age, and number of years in the U.S. |
| 11 | J. H. Kim | 2016 | Umbilical cord blood, newborns’ first urine | Perinatal | Generalized linear models | β and 95% CI | **(BMI z-score) β of natural log of ∑DEHP in each medium in maternal urine, -0.200 (-0.842, 0.442)**; β of natural log of ∑DEHP in cord blood, 0.110 (-3.053, 3.273); 0.114, (-2.197, 2.424) | maternal age, maternal BMI, gestational period, caesarean section, delivery experience, urinary creatinine levels, newborns’ sex, common log of ponderal index, and common log of triglyceride |
| 13 | D. W. Lee | 2020 | Prenatal maternal urine and urine of the participants | 6 y | Multivariate linear regression | β and 95% CI | **(BMI z-score) β of log_2_ ∑DEHP in prenatal maternal urine (-0.07 [-0.16, 0.02])**, and β of log_2_ ∑DEHP in children’s urine (-0.03 [-0.09, 0.14]); **β of log_2_ MnBP in prenatal maternal urine (-0.07 [95% CI: -0.17, 0.03])** and β of log_2_ MnBP in children’s urine -0.03 (95% CI: -0.15, 0.1) | maternal age, maternal education, and household income for the association between maternal phthalates, body composition indices of their children and urinary creatinine, and adjusted for maternal education, household income, energy intake per day, sex of the children, and urinary creatinine |
| 14 | M. M. Maresca | 2016 | Prenatal maternal urine | 5 y and 7 y | Generalized Estimating Equation  and principal component analysis | β and 95% CI | **(BMI z-score) β of natural log ∑DEHP were -0.09 (95% CI: -0.28, 0.11) among boys and -0.08 (95% CI: -0.25, 0.09) among girls**; **β of natural log MnBP were -0.22 (95% CI: -0.44, 0.00) among boys and 0.11 (95% CI: -0.12, 0.34) among girls**;  β of prenatal DEHP component score among boys (-0.00 [-0.24, 0.24]) and girls (-0.12 [-0.31, 0.08]); β of prenatal non-DEHP component score among boys (-0.30 [-0.54, -0.06]) and girls (0.13 [-0.14, 0.41]); | age, maternal pre-pregnancy obesity, birth weight, maternal race/ethnicity, maternal receipt of public assistance during pregnancy, urinary specific gravity, and urinary metabolite concentration component scores of children aged 3 and 5 y |
| 15 | K. G. Harley | 2017 | Prenatal maternal urine, two times | 5, 7, 9, 10.5, and 12 y | Generalized estimating euqations | β and 95% CI | **(BMI z-score) β of log_2_ ∑DEHP for BMI z-score at 5 year (0.05 [95% CI: -0.05, 0.16]).** β of log_2_ ∑DEHP for BMI z-score at 7 (0.08 [95% CI: -0.02, 0.18]), 9 (0.09 [95% CI: -0.01, 0.20]), 10.5 (0.09 [95% CI: -0.02, 0.19]) and 12 years (0.08 [95% CI: -0.03, 0.19)).  **β of log_2_ MnBP for BMI z-score at 5 year (0.09 [95% CI: 0.00, 0.19]).** β of log_2_ MnBP for BMI z-score at 7 (0.07 [95% CI: -0.01, 0.17]), 9 (0.06 [95% CI: -0.02, 0.14]), 10.5 (0.06 [95% CI: -0.03, 0.14]) and 12 years (0.06 [95% CI: -0.03, 0.15)). | maternal age, maternal education, marital status, years in United States prior to delivery, smoking during pregnancy, poverty status during pregnancy, child's  food insecurity at each time point, child’s fast food consumption at each time point, and prenatal bisphenol A exposure level |
| 17 | J. Shoaff | 2017 | up to two times prenatally and six times from 1 to 8 years of age | 8 y | Multiple informant model (Generalized estimating equation) | β and 95% CI | **(BMI z-score) β of log_10_ ∑DEHP at prenatal (-0.1 [95% CI: -0.4, 0.2])**; β of log_10_ ∑DEHP at 1 y, 2 y, 3 y, 4 y, 5 y, and 8 y (-0.4 [-0.8, 0.0], -0.2 [-0.6, 0.2], 0.1 [-0.3, 0.5], 0.1 [-0.3, 0.6], 0.4 [0.0, 0.9], and -0.1 [-0.4, 0.3], respectively); **β of log_10_ MnBP at prenatal (-0.1 [95% CI: -0.5, 0.4])**; | maternal age at delivery, race, marital status, insurance, income, education, parity, cotinine, depressive symptoms, mid pregnancy BMI, food security, fruit/vegetable and fish consumption, prenatal vitamin use, child’s sex, and child’s age at the visit |
| 21 | M. Vafeiadi | 2018 | Prenatal maternal urine and urine of the participants | 4–6 y | Generalized estimating equations | β and 95% CI | **(BMI z-score) β of log_10_ ∑DEHP in prenatal maternal urine (-0.21 [95% CI: -0.45, 0.03])** and children’s urine (-0.02 [95% CI: -0.27, 0.22])**; β of log_10_ sum of MnBP metabolites in maternal urine (-0.18 [95% CI: -0.41, 0.05])** and in children’s urine (0.15 [ 95% CI: -0.03, 0.34]) | sex, age, maternal age at delivery, parity, education, pre-pregnancy BMI, and smoking in pregnancy |
| 22 | D. Valvi | 2015 | Prenatal maternal urine at 1^st^ and 3^rd^ trimesters. | Birth to 6 mo., 1, 4, and 7 y | Generalized estimating equations | β and 95% CI | **(BMI z-score) β of log_2_ ∑DEHP (boys, -0.32 [95% CI: -0.64, -0.02]; girls, (0.21, [95% CI: -0.11, 0.53])** | sex, age, maternal country of origin, maternal age at delivery, maternal parity, maternal education, maternal social class, pre-pregnancy BMI, and smoking in pregnancy |
| 23 | M. Vrijheid | 2020 | 77 cases of prenatal exposure and 96 cases of childhood exposure | BMI z-score | Multivariate linear regression | β and 95% CI | (BMI z-score) β of IQR increase in ∑DEHP metabolites in maternal urine (-0.03 [-0.11, 0.05]; IQR 116.5 µg/g Cr) and in children’s urine (-0.04 [-0.13, 0.04]; IQR 75.3 µg/g Cr) | Enrolled cohort, sex, maternal BMI, maternal education, maternal age at conception, parity, parental country of origin, breastfeeding, and birth weight |
| 32 | K. Berger | 2021 | Prenatal maternal urine | 5 y | Bayesian hierachical model | β and 95% CI | β of ∑DEHP and MBP were -0.02 (95% CI: -0.12, 0.09), and 0.08 (0.00, 0.16) |  |
| 33 | L. Jiufeng | 2021 | Maternal urine in the 1st, 2nd and 3rd trimester of pregnancy | average BMI z-score of 6-, 12- and 24-month | Mixed linear model | β and 95% CI | **β of ∑DEHP, 0.134 (95% CI: -0.157, 0.425)** | maternal age, maternal height, pre-pregnancy body mass index, education levels, gestational age, pregnancy weight gain, parity, paternal height, and breastfeeding duration |
| 39 | A.Bowman | 2019 | Urine of the participants | 8–14y (Visit 1) and 9–17y (Visit 2) | Generalized estimating equation | β and 95% CI | **(BMI z-score) β of ∑DEHP in 2nd trimester prenatal maternal urine among boys and girls were 0.24 (95% CI: -0.13, 0.61), and -0.11 (95% CI: -0.45, 0.23), respectively. β of MnBP metabolites in 2^nd^ trimester prenatal maternal urine among boys and girls were -0.01 (95% CI: -0.29, 0.27) and -0.12 (95% Ci: -0.32, 0.08), respectively.** | specific gravity, maternal education, and age |

BMI, body mass index; CI, confidence interval; MEHHP, mono-(2-ethyl-5-hydroxy-hexyl) phthalate; MEOHP, mono-(2-ethyl-5-oxo-hexyl) phthalate; MECCP, Mono-2-ethyl-5-carboxypentyl phthalate; ∑DEHP, sum of di-2-ethylhexyl phthalate metabolites; MnBP, mono-n-butyl phthalate (MnBP); MBzP, Monobenzyl phthalate; IQR, interquartile range

Beta estimates used in the meta-analysis in bold

**Supplementary Table S6. Statistical significance of associations between prenatal phthalates exposure and BMI in children**

| **No** | **Author** | **∑phthalate** | **∑DEHP** | **MECCP** | **MEHHP** | **MEOHP** | **MnBP** | **MiBP** | **Remarks** |
| --- | --- | --- | --- | --- | --- | --- | --- | --- | --- |
| 1 | K. Agay-Shay | n.s. |  |  |  |  |  |  | BMI z-score at 7 years |
| 2 | Y. E. Berman | n.s. | n.s. | n.s. |  |  | n.s. | (+) | BMI z-score at 2-11 years |
| 3 | J. Botton |  | n.s. | n.s. | n.s. | n.s. | n.s. | n.s. | BMI at 5 years |
| 4 | J. P. Buckley |  | n.s. |  |  |  | n.s. | n.s. | BMI z-score at 4-7 years |
| 8 | A. L. Deierlein |  | n.s. |  |  |  | n.s. | n.s. | BMI at 6-8 years |
| 9 | B. C. Heggeseth |  |  | n.s. | n.s. | n.s. | n.s. | n.s. | BMI Trajectory Group |
| 11 | J. H. Kim |  | (+) |  | (+) | (+) |  |  | △BMI z-score form birth to 3 months |
| 13 | K. G. Harley |  | n.s. |  |  |  | n.s. | (+) | BMI z-score at 5 years |
| 14 | D. W. Lee |  | n.s. |  | n.s. | n.s. | n.s. |  | BMI z-score at 6 years |
| 15 | M. M. Maresca |  | n.s. |  |  |  |  |  | BMI z-score at ages 5 and 7 years |
| 17 | J. Shoaff |  | n.s. |  |  |  | n.s. | n.s. | BMI at 8 years |
| 21 | M. Vafeiadi |  | n.s. |  |  |  | n.s. | n.s. | BMI z-score at 4-6 years. Significant findings in girls |
| 22 | D. Valvi |  | n.s. |  |  |  |  |  | BMI z-score at 1, 4, and 7 years. Significant findings in boys |
| 23 | M. Vrijheid |  | n.s. | n.s. | n.s. | n.s. | n.s. | n.s. | BMI z-score at 6-11 years |
| 32 | K. Berger |  | n.s. |  |  |  | n.s. | n.s. | BMI z-score at 5years |
| 33 | L. Jiufeng |  | n.s. | n.s. | n.s. | n.s. |  |  | Children's average BMI z-score of 6-, 12-, and 24- months. |
| 39 | A. Bowman |  | n.s. | n.s. | n.s. | n.s. | (+) | (+) | BMI z-score at visit 1 (8–14 years) and at visit 2 (9–17 years) |

n.s., not statistically significant; (+), a statistically significant and positive association; (-), a statistically significant and negative association; ∑DEHP, sum of di-2-ethylhexyl phthalate metabolites; MEHHP, mono-(2-ethyl-5-hydroxy-hexyl) phthalate; MEOHP, mono-(2-ethyl-5-oxo-hexyl) phthalate; MECCP, Mono-2-ethyl-5-carboxypentyl phthalate; MnBP, mono-n-butyl phthalate (MnBP); MiBP, Monoisobutyl phthalate

**Supplementary Table S7. Description of studies on the association between prenatal exposure to phthalates and body fat percentage**

| **ID** |  | **First author** | **Year** | **Exposure assessment** | **Timing of outcome assessment** | **Statistical analysis** | **Estimates Type** | **Estimates** | **Adjustment variables** |
| --- | --- | --- | --- | --- | --- | --- | --- | --- | --- |
| 2 |  | Y. E. Berman | 2020 | Maternal urine in the 2^nd^ and 3^rd^ trimester of pregnancy | 1, 2, 3, 5, 8, 10, 14, 17, and 20 y | Linear mixed effect model | β and 95% CI or OR and 95% CI | (body fat percentage) ∑DEHP metabolites highest tertile (vs. lowest tertile), β 0.97 (0.84, 1.12); ∑DBP metabolites highest tertile (vs. lowest tertile), β 1.05 (0.91, 1.21) | age, gestational age at birth, birthweight, and maternal pre-pregnancy BMI |
| 5 |  | J. P. Buckley | 2016 | Prenatal maternal urine | 4 and 9 y | Linear mixed effect model | β and 95% CI | **(body fat percentage) β of natural log ∑DEHP (-0.89 [95% CI: -2.24, 0.47]) and β of natural log MnBP (-0.86 [95% CI: -0.37, 1.36])** | urine dilution and collection date, maternal race/ethnicity, age, education, work status, and smoking during pregnancy; maternal height and pre-pregnancy, BMI, adequacy of gestational weight gain, breastfeeding, child’s age in mos. and physical activity at follow-up, and child’s sex. |
| 13 |  | D. W. Lee | 2020 | Prenatal maternal urine and urine of the participants | 6 y | Multivariate linear regression | β and 95% CI | (body fat percentage) **β of log_2_ ∑DEHP at prenatal (-0.05 [95% CI:-0.09, -0.02])** and **β of log_2_ MnBP at prenatal (0.01 [95% CI: -0.31, 0.33])** | maternal age, maternal education, and household income for the association between maternal phthalates, body composition indices and urinary creatinine of the children. Parameters were adjusted for maternal education, household income, energy intake per day, sex of the children, and urinary creatinine |
| 14 |  | M. M. Maresca | 2016 | Prenatal maternal urine | 5 and 7 y | Generalized Estimating Equation | β and 95% CI | (body fat percentage) **β of natural log ∑DEHP among boys (-0.39 [95% CI: -1.57, 0.79]) and girls (-0.13 [95% CI: -1.09, 0.84]); β of natural log MnBP among boys (-1.05 [95% CI: -2.26, 0.15]), and girls (-0.52 [95% CI: -0.72, 1.76])** | age, maternal pre-pregnancy obesity, birth weight, maternal race/ethnicity, maternal receipt of public assistance during pregnancy, urinary specific gravity, and urinary metabolite concentration component scores of children at 3 and 5 years of age |
| 15 |  | K. G. Harley | 2017 | Prenatal maternal urine, two times | 5, 7, 9, 10.5, and 12 y | Generalized estimating euqations | β and 95% CI | (body fat percentage) **β of log_2_ ∑DEHP at 9 (1.0 [95% CI: -0.2, 2.2])**, 10.5 (1.0 [95% CI: -0.2, 2.2]), and 12 year (1.1 [95% CI: -0.2, 2.4]); **β of log_2_ MnBP at 9 (0.9 [95% CI: -0.1, 1.9])**, 10.5 (1.0 [95% CI: 0.0, 1.9]), and 12 year (0.7 [95% CI: -0.4, 1.8]) | maternal age, maternal education, marital status, years in United States prior to delivery, smoking during pregnancy, poverty status during pregnancy, child's  food insecurity at each time point, child’s fast food consumption at each time point, and prenatal bisphenol A |
| 17 |  | J. Shoaff | 2017 | up to two times prenatally and six times from 1 to 8 years of age | 8 y | Multiple informant model (Generalized estimating equation) | β and 95% CI | (body fat percentage) **β of log_10_ ∑DEHP at prenatal (0.5 [95% CI: -1.4, 2.3])** | maternal age at delivery, race, marital status, insurance, income, education, parity, urinary cotinine levels, depressive symptoms, mid pregnancy BMI, food security, fruit/vegetable and fish consumption, prenatal vitamin use, child’s sex, and child’s age in months at the visit |
| 27 |  | A. Zettergren | 2021 | Urine of the participants at 4 years of age | 24 y | Generalized estimating equations and multivariate linear regression | β and 95% CI | (body fat percentage) **β of natural log ∑DEHP (1.62 [95% CI: -0.97, 4.20]) and β of natural log MnBP (-0.77, [95% CI: -3.47, 1.94])** | sex, maternal smoking during pregnancy, socioeconomic status, breastfeeding duration, physical activity, smoking, and urinary cotinine levels. |

BMI, body mass index; CI, confidence interval; MEHHP, mono-(2-ethyl-5-hydroxy-hexyl) phthalate; MEOHP, mono-(2-ethyl-5-oxo-hexyl) phthalate; MECCP, Mono-2-ethyl-5-carboxypentyl phthalate; ∑DEHP, sum of di-2-ethylhexyl phthalate metabolites; MnBP, mono-n-butyl phthalate (MnBP); MBzP, Monobenzyl phthalate; IQR, interquartile range

Beta estimates used in the meta-analysis in bold

**Supplementary Table S8. Statistical significance of associations between prenatal phthalates exposure and percent fat mass in children**

| **No** | **Author** | **∑phthalate** | **∑DEHP** | **MECCP** | **MEHHP** | **MEOHP** | **MnBP** | **MiBP** | **Remarks** |
| --- | --- | --- | --- | --- | --- | --- | --- | --- | --- |
| 2 | Y. E. Berman | n.s. | n.s. | (+) |  |  | n.s. | n.s. | Percent fat mass at 2-11 years |
| 5 | J. P. Buckley |  | n.s. | n.s. |  |  | n.s. |  | Percent fat mass at 4-7 years |
| 13 | D. W. Lee |  | n.s. |  | n.s. | n.s. | n.s. |  | Percent fat mass at 6 years |
| 14 | M. M. Maresca |  | n.s. |  |  |  |  |  | Percent fat mass at 5 and 7 years |
| 15 | K. G. Harley |  | (+) |  |  |  | n.s. | n.s. | Percent fat mass at 5 years |
| 17 | J. Shoaff |  | n.s. |  |  |  | n.s. | n.s. | Percent fat mass at 8 years |
| 27 | A. Zettergren |  | n.s. | n.s. | n.s. | n.s. | n.s. |  | Percent fat mass at 24 years |

n.s., not statistically significant; (+), a statistically significant and positive association; (-), a statistically significant and negative association; ∑DEHP, sum of di-2-ethylhexyl phthalate metabolites; MEHHP, mono-(2-ethyl-5-hydroxy-hexyl) phthalate; MEOHP, mono-(2-ethyl-5-oxo-hexyl) phthalate; MECCP, Mono-2-ethyl-5-carboxypentyl phthalate; MnBP, mono-n-butyl phthalate (MnBP); MiBP, Monoisobutyl phthalate

**Supplementary Table S9. Description of studies on the association between prenatal exposure to phthalates and other body composition indices**

| **ID** | **First author** | **Year** | **Exposure assessment** | **Timing of outcome assessment** | **Statistical analysis** | **Estimates Type** | **Outcome variables** | **Estimates** | **Adjustment variables** |
| --- | --- | --- | --- | --- | --- | --- | --- | --- | --- |
| 2 | Y. E. Berman | 2020 | Maternal urine in the 2nd and 3rd trimester of pregnancy | 1, 2, 3, 5, 8, 10, 14, 17 and 20 y | Linear mixed effect model | β and 95% CI or OR and 95% CI | Height, BMI, DXA (total fat %, total fat mass [g], total lean mass [g]) | (Deviation from mid-parental height at 20 y z-score) ∑DEHP metabolites highest tertile (vs. lowest tertile), β 0.08 (95% CI: -0.21, 0.37); ∑DBP metabolites highest tertile (vs. lowest tertile), β 0.10 (95% CI: -0.19, 0.40) (total fat %) ∑DEHP metabolites highest tertile (vs. lowest tertile), OR 0.97 (95% CI: 0.84, 1.12); ∑DBP metabolites highest tertile (vs. lowest tertile), OR 1.05 (95% CI: 0.91, 1.21) | age, gestational age at birth, birthweight, and maternal pre-pregnancy BMI |
| 4 | J. P. Buckley | 2016 | Prenatal maternal urine | 4–9 y | Linear mixed effect model | β and 95% CI or OR and 95% CI | BMI z-score and overweight/obesity (BMI >= 85th percentile) | (Overweight/obese) OR for standard deviation increase in natural log ∑DEHP metabolites, MnBP and MiBP (0.87 [95% CI: 0.53, 1.4], 1.0 [95% CI: 0.51, 2.0], and 0.84 [95% CI: 0.44, 1.6], respectively) | cohort, maternal race/ethnicity, maternal age at delivery, maternal education, maternal work status during pregnancy, maternal pre-pregnancy BMI, gestational weight gain, maternal smoking during pregnancy, calendar date of urine collection, and parity. |
| 13 | D. W. Lee | 2020 | Prenatal maternal urine and urine of the participants | 6 y | Multivariate linear regression | β and 95% CI | BMI z-score, percentage of fat mass, fat mass index, percentage of skeletal muscle mass, skeletal muscle index | (Skeletal muscle index) β of two-fold increase of ∑DEHP in prenatal maternal urine and in children’s urine (-0.05, [95% CI: -0.09, -0.02], and 0.003 [95% CI: -0.05, 0.053], respectively); MnBP in prenatal maternal urine and in children’s urine (-0.05 [95% CI: -0.09, -0.005], and -0.04 [95% CI: -0.10, 0.01], respectively) | maternal age, maternal education, and household income for the association between maternal phthalates, body composition indices of their children and urinary creatinine, and adjusted for maternal education, household income, energy intake per day, sex of the child, and urinary creatinine |
| 14 | M. M. Maresca | 2016 | Prenatal maternal urine | 5 y and 7 y | Generalized Estimating Equation | β and 95% CI | BMI z-score at 5 y and 7 y, percent of fat mass at 7 y, FMI at 7 y, WC at 7 y | (FMI) β of ln-transformed ∑DEHP, and MnBP among girls (-0.13 [95% CI: -0.19, 0.84], and -0.52 [95% CI: -0.72, 1.76], respectively), among boys (-0.39 [95% CI: -1.57, 0.79]), and -1.05 [95% CI: -2.26, 0.15]), respectively)  (WC) β of ln-transformed ∑DEHP, and MnBP among girls (-0.13 [95% CI: -1.37, 1.12], and 0.85 [95% CI: -0.76, 2.47], respectively), among boys (-0.65 [95% CI: -2.16, 0.87]), and -1.34 [95% CI: -2.91, 0.23]), respectively) | age, maternal pre-pregnancy obesity, birth weight, maternal race/ethnicity, maternal receipt of public assistance during pregnancy, urinary specific gravity, and urinary metabolite concentration component scores of children aged 3 and 5 y |
| 15 | K. G. Harley | 2017 | Prenatal maternal urine, two times | 5, 7, 9, 10.5, and 12 y | Generalized estimating euqations | β and 95% CI | WC z-score | (WC z-score) β of 2-fold increase in ∑DEHP was associated with WC z-score at 5, 7, 9, 10.5, and 12 year as 0.14 (95% CI: 0.05, 0.23), 0.00 (95% CI: -0.08, 0.09), 0.00 (95% CI: -0.09, 0.09), 0.10 (95% CI: 0.00, 0.19) and 0.09 (95% CI: -0.01, 0.20). β of 2-fold increase in MnBP was associated with WC z-score at 5, 7, 9, 10.5, and 12 year as 0.07 (95% CI: -0.01, 0.14), 0.12 (95% CI: 0.05, 0.19), 0.12 (95% CI: 0.05, 0.19), 0.05 (95% CI: -0.03, 0.12), and 0.04 (95% CI: -0.04, 0.13). | maternal age, maternal education, marital status, years in U.S. prior to delivery, smoking during pregnancy, poverty status during pregnancy, child's  food insecurity at each time point, child’s fast food consumption at each time point, and prenatal bisphenol A |
| 17 | J. Shoaff | 2017 | up to two times prenatally and six times from 1 to 8 years of age | 8 y | Multiple informant model (Generalized estimating equation) | β and 95% CI | BMI z-score, WC, body fat percent | (body fat percentage) β of ∑DEHP at prenatal, 1 y, 2 y, 3 y, 4 y, 5 y, and 8 y (0.5 [95% CI: -1.4, 2.3], -2.7 [95% CI: -4.8, -0.5], -1.4 [95% CI: -3.9, 1.2], 0.9 [95% CI: -1.5, 3.3], 1.3 [95% CI: -1.2, 3.9], 2.9 [95% CI: 0.3, 5.5], and -0.6 [95% CI: -2.8, 1.6], respectively); | maternal age at delivery, race, marital status, insurance, income, education, parity, cotinine, depressive symptoms, mid pregnancy BMI, food security, fruit/vegetable and fish consumption, prenatal vitamin use, child’s sex, and child’s age at the 8-year visit |
| 20 | Y. A. Tsai | 2016 | Estimated the total daily intake of DEHP, and urine of the participants | When participants were examined | Logistic regression | OR and 95% CI | Weight percentile and Height percentile above 50^th^ percentile | (Weight) OR of estimated phthalates dietary intake (>median vs. <= median), 0.07 (95% CI: 0.02, 0.19); (Height) OR of estimated dietary intake (>median vs. <= median), 0.30, (95% CI: 0.12, 0.75) | sex, maternal age at pregnancy |
| 22 | D. Valvi | 2015 | Prenatal maternal urine at 1st and 3rd trimester. | Birth to 6 months., 1, 4, and 7 years of age | Generalized estimating equations | β and 95% CI | BMI z-score, weight gain z-score (0–6 months) | (Weight gain z-score 0–6 months) β of one unit increase in ∑DEHP metabolites in girls (0.26 [95% CI: -0.13, 0.65]) and in boys (-0.36 [95% CI: -0.70, -0.01]) | sex, age, maternal country of origin, maternal age at delivery, maternal parity, maternal education, maternal social class, pre-pregnancy BMI, and smoking in pregnancy |
| 34 | N. Nidens | 2021 | Prenatal maternal urine | 2y | Multivariate linear regression | β and 95% CI | Weight gain (%) first 2 years of life | β of ∑HMWP, -0.69 (95% CI: -14.01, 12.62) | child sex, birth weight, maternal age, pre-pregnancy BMI, socioeconomic status, and smoking status |
| 39 | A.Bowman | 2019 | Urine of the participants | 8–14y (Visit 1) and 9–17y (Visit 2) | Generalized estimating equation | β and 95% CI | WC, skinfold thickness | (WC) β of ∑DEHP in 2nd trimester prenatal maternal urine among boys and girls were 1.63 (95% CI: -1.06, 4.32), and -0.46 (95% CI: -3.57, 2.66), respectively (skinfold thickness) β of ∑DEHP in 2nd trimester prenatal maternal urine among boys and girls were 0.56 (95% CI: -2.87, 4.00), and -1.44 (95% CI: -4.77, 1.89), respectively | specific gravity, maternal education, and age |

BMI, body mass index; CI, confidence interval; MEHHP, mono-(2-ethyl-5-hydroxy-hexyl) phthalate; MEOHP, mono-(2-ethyl-5-oxo-hexyl) phthalate; MECCP, Mono-2-ethyl-5-carboxypentyl phthalate; ∑DEHP, sum of di-2-ethylhexyl phthalate metabolites; MnBP, mono-n-butyl phthalate (MnBP); MBzP, Monobenzyl phthalate; WC, waist circumference; DXA, dual-energy X-ray absorptiometry; IQR, interquartile range; OR, odds ratio; ∑HMWP, sum of high molecular weight phthalate metabolites

**Supplementary Table S10. Description of studies on the association between postnatal exposure to phthalates and BMI**

| **ID** | **First author** | **Year** | **Exposure assessment** | **Timing of outcome assessment** | **Statistical analysis** | **Esti-mates Type** | **Estimates** | **Adjustment variables** |
| --- | --- | --- | --- | --- | --- | --- | --- | --- |
| 6 | M. C. Buser | 2014 | Urine of the participants | children and adolescents aged 6–19 y, adults >= 20 years of age | Multivariate linear regression and logistic regression | β and 95% CI or OR and 95% CI | (BMI z-score) β of One unit increase in natural log-transformed sum of DEHP metabolites and LMW metabolites (-0.01 [95% CI: -0.06, 0.04] and 0.07 [95% CI: 0.02, 0.13]) (Obesity) OR of one unit increase in natural log-transformed sum of DEHP metabolites and LMW metabolites (1.04 [95% CI: 0.89, 1.20], and 1.02 [95% CI: 0.90, 1.17]) | urinary creatinine, sex, poverty-income ratio, parental education, serum cotinine, age, race/ethnicity category, caloric intake, and watching television |
| 7 | C. H. Chang | 2020 | Urine of the participants | 5 y | Multivariate linear regression | β and 95% CI | (BMI z-score) β of IQR increase in sum of DEHP metabolites in maternal urine (-0.03 [95% CI: -0.11, 0.05]; IQR 116.5 µg/g Cr) and in children’s urine (-0.04 [95% CI: -0.13, 0.04]; IQR 75.3 µg/g Cr) | cohort, sex, maternal BMI, maternal education, maternal age at conception, parity, parental country of origin, breastfeeding, and birth weight |
| 8 | A. L. Deierlein | 2016 | Urine of the participants at the baseline (6–8 y) | 3 times until the last visit when girls were on average 14 y old (11–16 y) | Multivariate linear regression, WQS | β and 95% CI | (BMI z-score) β of MiBP (highest quartile vs. lowest quartile), 0.10 (95% CI: -0.07, 0.27) | age, sex, race, educational levels, family income-to-poverty ratio, caloric intake, serum cotinine, and log-transformed creatinine |
| 10 | J. W. Hou | 2015 | Urine of the participants | When assessing phthalates exposure (6.5–8.5 y) | Generalized estimating equations and multivariate linear regression | β and 95% CI | (BMI) β of one unit increase in log-transformed phthalate metabolite concentrations of sum of DEHP (1.24 [95% CI: -0.31, 2.79]) and MnBP (-0.11, [95% CI: -1.75, 1.53]) (WC) β of one unit increase in log-transformed phthalate metabolite concentrations of sum of DEHP (3.44 [95% CI: -0.45, 7.33] and MnBP (0.36, [95% CI: -3.77, 4.48]) (Body fat %) β of one unit increase in log-transformed phthalate metabolite concentrations of sum of DEHP (1.62 [95% CI: -0.97, 4.20]) and MnBP (-0.77, [95% CI: -3.47, 1.94]) (Trunk fat %) β of one unit increase in log-transformed phthalate metabolite concentrations of sum of DEHP (1.61 [95% CI: -1.26, 4.49]) and MnBP (95% CI: -1.05 [-4.05, 1.95]) | Sex, maternal smoking during pregnancy, socioeconomic status, breastfeeding duration, physical activity, smoking, and urinary cotinine. |
| 12 | S. H. Kim | 2018 | Urine of the participants | When assessing phthalates exposure (6–13 y) | Multivariate linear regression and logistic regression | β and 95% CI or OR and 95% CI | (BMI z-score) β of One unit increase in natural log-transformed sum of DEHP metabolites and LMW metabolites (-0.01 [95% CI: -0.06, 0.04] and 0.07 [95% CI: 0.02, 0.13]) (Obesity OR) OR of One unit increase in natural log-transformed sum of DEHP metabolites and LMW metabolites (1.04 [95% CI: 0.89, 1.20], and 1.02 [95% CI: 0.90, 1.17]), -0.01 [95% CI: -0.06, 0.04] and 0.07 [95% CI: 0.02, 0.13]) | urinary creatinine, sex, poverty-income ratio, parental education, serum cotinine, age, race/ethnicity category, caloric intake, and watching television |
| 16 | T. Saengkaew | 2017 | Urine of the participants | When assessing phthalates exposure | Multivariate linear regression | β and 95% CI | (BMI z-score) β of IQR increase in sum of DEHP metabolites in maternal urine (-0.03 [95% CI: -0.11, 0.05]; IQR 116.5 µg/g Cr) and in children’s urine (-0.04 [95% CI: -0.13, 0.04]; IQR 75.3 µg/g Cr) | Cohort, sex, maternal BMI, maternal education, maternal age at conception, parity, parental country of origin, breastfeeding, and birth weight |
| 17 | J. Shoaff | 2017 | up to two times prenatally and six times from 1 to 8 years of age | 8 y | Multivariate linear regression, WQS | β and 95% CI | (BMI z-score) β of MiBP (Highest quartile vs. lowest quartile), 0.10 (95% CI: -0.07, 0.27) | age, sex, race, educational levels, family income-to-poverty ratio, caloric intake, serum cotinine, and log-transformed creatinine |
| 19 | L. Trasande | 2013 | Urine of the participants | When assessing phthalate exposure | Generalized estimating equations and multivariate linear regression | β and 95% CI | (BMI) β of one unit increase in log-transformed phthalate metabolite concentrations of sum of DEHP (1.24 [95% CI: -0.31, 2.79]) and MnBP (-0.11, [95% CI: -1.75, 1.53]) (WC) β of one unit increase in log-transformed phthalate metabolite concentrations of sum of DEHP (3.44 [95% CI: -0.45, 7.33] and MnBP (0.36, [95% CI: -3.77, 4.48]) (Body fat %) β of one unit increase in log-transformed phthalate metabolite concentrations of sum of DEHP (1.62 [95% CI: -0.97, 4.20]) and MnBP (-0.77, [95% CI: -3.47, 1.94]) (Trunk fat %) β of one unit increase in log-transformed phthalate metabolite concentrations of sum of DEHP (1.61 [95% CI: -1.26, 4.49]) and MnBP (-1.05 [95% CI: -4.05, 1.95]) | sex, maternal smoking during pregnancy, socioeconomic status, breastfeeding duration, physical activity, smoking, and urinary cotinine levels. |
| 23 | M. Vrijheid | 2020 | 77 cases of prenatal exposure and 96 cases of childhood exposure including exposure to air pollutants, built environments, and biomarkers of chemical pollutants. | BMI z-score (age-and-sex standardized z-scores) | Logistic regression | OR and 95% CI | (Obesity vs normal) OR of MBP (highest quartile vs. lowest quartile) among boys, 5.768 (95% CI: 1.622, 20.515); OR of sum of DEHP metabolites (highest quartile vs. lowest quartile) among girls, 0.078 (95% CI: 0.008, 0.791) | socioeconomic level, physical activity, dietary nutriment intake and puberty onset, phthalate metabolite concentrations. |
| 24 | B. Wu | 2020 | Urine of the participants | When assessing phthalate exposure | Multivariate linear regression and logistic regression | β and 95% CI or OR and 95% CI | (BMI z-score) β of One unit increase in natural log-transformed sum of DEHP metabolites and LMW metabolites (-0.01 [95% CI: -0.06, 0.04] and 0.07 [95% CI: 0.02, 0.13]) (Obesity OR) OR of One unit increase in natural log ∑DEHP metabolites and LMW metabolites (1.04 [95% CI: 0.89, 1.20], and 1.02 [95% CI: 0.90, 1.17]), -0.01 [95% CI: -0.06, 0.04] and 0.07 [95% CI: 0.02, 0.13]) | urinary creatinine, sex, poverty-income ratio, parental education, serum cotinine, age, race/ethnicity category, caloric intake, and watching television |
| 27 | A. Zettergren | 2021 | Urine of the participants at 4 years of age | 24 y | Multivariate linear regression | β and 95% CI | (BMI z-score) β of IQR increase in sum of DEHP metabolites in maternal urine (-0.03 [95% CI: -0.11, 0.05]; IQR 116.5 µg/gCr) and in children’s urine (-0.04 [95% CI: -0.13, 0.04]; IQR 75.3 µg/g Cr) | cohort, sex, maternal BMI, maternal education, maternal age at conception, parity, parental country of origin, breastfeeding, and birth weight |
| 28 | Y. Zhang | 2014 | Urine of the participants at 8–13y | When assessing phthalates exposure | Multivariate linear regression, WQS | β and 95% CI | (BMI z-score) β of MiBP (highest quartile *vs.* lowest quartile), 0.10 (95% CI: -0.07, 0.27) | age, sex, race, educational levels, family income-to-poverty ratio, caloric intake, serum cotinine, and log-transformed creatinine |
| 29 | M.M. Amin | 2018 | Urine of participants at 6–18y | When assessing phthalates exposure | Multivariate linear regression | β and *p* value | (BMI z-score) β (*p*-value*)* of MEOHP, MEHHP, MEHP, MBzP, and MnBP were 0.17 (0.005), 0.3 (<0.001), 0.23 (<0.001), 0.18 (0.002), and 0.22 (<0.001). | Sex, age, and physical activity |
| 30 | J. Ashley-Martin | 2021 | Urine of the participants at 2–5y | 2–5 y | Multivariate linear regression | β and 95% CI | (BMI z-score) β of ∑DEHP and ∑DiBP (highest tertile vs. lowest tertile) were 0.019 (95% CI: -0.391, 0.429), and 0.475 (95% CI: 0.068, 0.883), respectively | maternal postnatal body mass index, household income, maternal age, and specific gravity |
| 31 | S. Ding | 2021 | Urine of the participants at 16–19y | 16–19 y | Multivariate linear regression | β and 95% CI | (BMI) β of ∑DEHP, 0.024 (95% CI: -0.001, 0.050) | sex. Age. residential area, average household income, physical exercise, and urinary creatinine level |
| 35 | J. On | 2021 | Urine of the participants at 5–16y | 5–16 y | Multivariate linear regression | β and 95% CI | (BMI percentile) β of MEHHP and MEOHP were 0.839 (95% CI: 0.349, 1.328), and -0.937 (-1.474, and -0.401) | age |
| 36 | C. Silva | 2021 | Urine of the participants at 6y | 6y and 10 y | Multivariate linear regression | β and 95% CI | (BMI z-score, 6y) β of IQR increase of ∑DEHP IQR increase), 0.06 (95% CI: -0.05, 0.16) (BMI z-score, 10y) β of IQR increase of ∑DEHP, 0.10 (95% CI: -0.04, 0.17) | maternal educational level and child sex, age (except for sex- and age-adjusted BMI z scores), diet quality score, and television-watching time |
| 37 | E. E. Hatch | 2008 | Urine of the participants | 6–19y | Multivariate linear regression | β and 95% CI | (BMI) β of MEHHP (the highest quartile vs. the lowest quartile) among boys in 6–11 y and 12–19y were 0.42 (95% CI: -1.09, 1.92) and 1.00 (-0.69, 2.69), respectively (BMI) β of MEHHP (the highest quartile vs. the lowest quartile) among girls in 6–11 y and 12–19y were 0.54 (95% CI: -1.50, 2.57) and 0.74 (-1.18, 2.65), respectively | age, creatinine, height, race/ethnicity, socioeconomic status, % of daily calories from total fat, daily servings of dairy, daily servings of fruit and vegetables, METS/month, and TV/video/computer use |
| 38 | H. Wang | 2013 | Urine of the participants | 8–15y | Multivariate linear regression | β and 95% CI | (BMI) β of ∑DEHP, 0.037 (95% CI: 0.006, 0.067) | age, sex, and urine specific gravity |

BMI, body mass index; OR, odds ratio; CI, confidence interval; MEHHP, mono-(2-ethyl-5-hydroxy-hexyl) phthalate; MEOHP, mono-(2-ethyl-5-oxo-hexyl) phthalate; MECCP, Mono-2-ethyl-5-carboxypentyl phthalate; ∑DEHP, sum of di-2-ethylhexyl phthalate metabolites; MnBP, mono-n-butyl phthalate (MnBP); MBzP, Monobenzyl phthalate; LMW, low molecular weight; WC, waist circumference; IQR, interquartile range; WQS, weight quantile sum; MiBP, monoisobutyl phthalate

**Supplementary Table S11. Statistical significance of associations of phthalates exposure at children with BMI, obese, and obesity in children**

| **No** | **Author** | **∑phthalate** | **∑DEHP** | **MECCP** | **MEHHP** | **MEOHP** | **MnBP** | **MiBP** | **Remarks** |
| --- | --- | --- | --- | --- | --- | --- | --- | --- | --- |
| 6 | M. C. Buser |  | n.s. | n.s. | n.s. | n.s. | n.s. | n.s. | Obesity (BMI z-score >= 95th percentile) and overweight (BMI z-score >= 85th percentile) at 6-19 years old |
| 7 | C. H. Chang |  | n.s. |  |  |  | n.s. | n.s. | MEP and MBzP were significantly and positively associated with BMI of children (mean age=9.9 y) |
| 8 | A. L. Deierlein |  | n.s. | n.s. | n.s. | (+) | n.s. | n.s. | Predicted differences in BMI at ages 7-13 years according to quartiled phthalates at 6-8 years |
| 10 | J. W. Hou |  | n.s. | n.s. | (+) | n.s. | n.s. | n.s. | Overweight defined by BMI by phthalates (<25th, 25-75th, and >=75th percentile) |
| 12 | S. H. Kim |  | n.s. | n.s. | (+) | n.s. |  |  | BMI percentile by percentage fractions of DEHP metabolites |
| 16 | T. Saengkaew |  |  |  |  |  | n.s. |  | No differences in MMP and MnBP between normal weight children (n=70) and overweight/obesity children (n=85) |
| 17 | J. Shoaff |  | n.s. |  |  |  | n.s. | n.s. | Multiple informants model estimates of difference in BMI z-score per 10-fold increase in urinary phthalate metabolite concentration during pregnancy and childhood at 1, 2, 3, 4, 5, and 8 years of age |
| 19 | L. Trasande |  | n.s. |  |  |  |  |  | Among non-Hispanic black children, there was significnat association of MEP and MBP with obesity |
| 23 | M. Vrijheid |  | n.s. | n.s. | n.s. | n.s. | n.s. | n.s. | Obesity defined by BMI z-score |
| 24 | B. Wu |  |  |  |  |  |  | (+) | MEP and MiBP were positvely associated with obesity in children aged 6-19 in NHANES 2005-2010 |
| 27 | A. Zettergren |  | n.s. | n.s. | n.s. | n.s. | n.s. |  | DiNP metabolites were associated with overweight/obesity |
| 28 | Y. Zhang | (+) |  |  | (+) |  | (+) |  | Associations were positive for MBP and sum of LMP with both BMI z-score and fat distribution in boys >10 years of age, and negative for sum of MEHP with fat distribution in girls <10 years of age. |
| 29 | M.M. Amin |  |  |  | (+) | (+) | (+) |  | BMI z-score as a continuous variable |
| 30 | J. Ashley-Martin |  | n.s. |  |  |  |  |  | There was no statisitically significnace between prenatal exposure to mixtures of phthalates, parabens, and other phenols and obesity |
| 31 | S. Ding |  | n.s. | n.s. | n.s. | n.s. |  |  | Among boys, MECPP, MEHHP, MEOHP in prenatal maternal urine were significantly associated with children's average BMI z-score of 6-, 12-, and 24- months. |
| 35 | J. On |  |  |  | n.s. | n.s. |  |  |  |
| 36 | C. Silva |  | n.s. |  |  |  |  |  | DNOP (di-n-octyl phthalate) metabolites are associated with overweight and an adverse cardiovascular profile in childhood. |
| 37 | E. E. Hatch |  |  |  | n.s. | n.s. | n.s. |  | BMI and WC increased with MEP quartile in adolescent girls |
| 38 | H. Wang |  | (+) | n.s. | n.s. | n.s. | (+) | (+) | Some phthalate exposures were associated with BMI or WC in Chinese school children |

n.s., not statistically significant; (+), a statistically significant and positive association; (-), a statistically significant and negative association; ∑DEHP, sum of di-2-ethylhexyl phthalate metabolites; MEHHP, mono-(2-ethyl-5-hydroxy-hexyl) phthalate; MEOHP, mono-(2-ethyl-5-oxo-hexyl) phthalate; MECCP, Mono-2-ethyl-5-carboxypentyl phthalate; MnBP, mono-n-butyl phthalate (MnBP); MiBP, Monoisobutyl phthalate; LMP, low molecular weight phthalate

**Supplementary Table S12. Description of studies on the association between postnatal exposure to phthalates and body indices other than BMI**

| **ID** | **First author** | **Year** | **Exposure assessment** | **Timing of outcome assessment** | **Statistical analysis** | **Estimates Type** | **Estimates** | **Adjustment variables** |
| --- | --- | --- | --- | --- | --- | --- | --- | --- |
| 7 | C. H. Chang | 2020 | Urine of the participants | 5 y | Logistic regression | OR and 95% CI | (CDGP) OR (the highest tertile vs. the lowest tertile) of MnBP, MEP, MEHP, and total phthalates were 8.30 (95% CI: 1.97, 34.44), 5.43 (95% CI: 2.02, 14.55), 3.83 (95% CI: 1.59, 8.68), and 9.09 (95% CI: 3.16, 26.31), respectively. | age, BMI, other phthalate metabolites |
| 10 | J. W. Hou | 2015 | Urine of the participants | When assessing phthalates exposure (6.5–8.5 y) | Generalized estimating equations and multivariate linear regression | β and 95% CI | (WC) β of one unit increase in log-transformed phthalate metabolite concentrations of sum of DEHP (3.44 [95% CI: -0.45, 7.33] and MnBP (0.36, [95% CI: -3.77, 4.48]) (Body fat %) β of one unit increase in log-transformed phthalate metabolite concentrations of sum of DEHP (1.62 [95% CI: -0.97, 4.20]) and MnBP (-0.77, [95% CI: -3.47, 1.94]) (Trunk fat %) β of one unit increase in log-transformed phthalate metabolite concentrations of sum of DEHP (1.61 [95% CI: -1.26, 4.49]) and MnBP (-1.05 [95% CI: -4.05, 1.95]) | sex, maternal smoking during pregnancy, socioeconomic status, breastfeeding duration, physical activity, smoking, and urinary cotinine. |
| 16 | T. Saengkaew | 2017 | Urine of the participants | When assessing phthalates exposure | Logistic regression | OR and 95% CI | (Obesity vs normal) OR of MBP (highest quartile vs. lowest quartile) among boys, 5.768 (95% CI: 1.622, 20.515); OR of sum of DEHP metabolites (highest quartile vs. lowest quartile) among girls, 0.078 (95% CI: 0.008, 0.791) | socioeconomic level, physical activity, dietary nutriment intake and puberty onset, phthalate metabolite concentrations. |
| 17 | J. Shoaff | 2017 | Up to two times prenatally and six times from 1 to 8 years of age | 8 y | Logistic regression | OR and 95% CI | (CDGP) OR (highest tertile vs. lowest tertile) of MBP, MEP, MEHP, and total phthalates were 8.30 (95% CI: 1.97, 34.44), 5.43 (95% CI: 2.02, 14.55), 3.83 (95% CI: 1.59, 8.68), and 9.09 (95% CI: 3.16, 26.31), respectively. | age, BMI, other phthalates metabolites |
| 18 | A. Smerieri | 2015 | Urine of the participants | When assessing phthalate exposure | Generalized estimating equations and multivariate linear regression | β and 95% CI | (WC) β of one unit increase in log-transformed phthalate metabolite concentrations of sum of DEHP (3.44 [95% CI: -0.45, 7.33] and MnBP (0.36, [95% CI: -3.77, 4.48]) (Body fat %) β of one unit increase in log-transformed phthalate metabolite concentrations of sum of DEHP (1.62 [95% CI: -0.97, 4.20]) and MnBP (-0.77, [95% CI: -3.47, 1.94]) (Trunk fat %) β of one unit increase in log-transformed phthalate metabolite concentrations of sum of DEHP (1.61 [95% CI: -1.26, 4.49]) and MnBP (-1.05 [95% CI: -4.05, 1.95]) | sex, maternal smoking during pregnancy, socioeconomic status, breastfeeding duration, physical activity, smoking, and urinary cotinine. |
| 26 | C. Xie | 2015 | Urine of the participants | When assessing phthalate exposure | Logistic regression | OR and 95% CI | (CDGP) OR (the highest tertile vs. the lowest tertile) of MBP, MEP, MEHP, and total phthalates were 8.30 (95% CI: 1.97, 34.44), 5.43 (95% CI: 2.02, 14.55), 3.83 (95% CI: 1.59, 8.68), and 9.09 (95% CI: 3.16, 26.31), respectively. | age, BMI, other phthalate metabolites |
| 27 | A. Zettergren | 2021 | Urine of the participants at 4 years of age | 24 y | Generalized estimating equations and multivariate linear regression | β and 95% CI | (WC) β of one unit increase in log-transformed phthalate metabolite concentrations of sum of DEHP (3.44 [95% CI: -0.45, 7.33] and MnBP (0.36, [95% CI: -3.77, 4.48]) (Body fat %) β of one unit increase in log-transformed phthalate metabolite concentrations of sum of DEHP (1.62 [95% CI: -0.97, 4.20]) and MnBP (-0.77, [95% CI: -3.47, 1.94]) (Trunk fat %) β of one unit increase in log-transformed phthalate metabolite concentrations of sum of DEHP (1.61 [95% CI: -1.26, 4.49]) and MnBP (-1.05 [95% CI: -4.05, 1.95]) | sex, maternal smoking during pregnancy, socioeconomic status, breastfeeding duration, physical activity, smoking, and urinary cotinine. |
| 29 | M.M. Amin | 2018 | Urine of participants | When assessing phthalates exposure | Multivariate linear regression | β and *p* value | (WC) β (*p*-value*)* of MEOHP, MEHHP, MEHP, MBzP, and MiBP were 0.19 (0.003), 0.39 (<0.001), 0.37 (<0.001), 0.22 (<0.001), and 0.29 (<0.001). | sex, age, and physical activity |
| 31 | S. Ding | 2021 | Urine of the participants at 16–19y | 16–19 y | Multivariate linear regression | β and 95% CI | (WHR) β of ∑DEHP, 0.012 (95% CI: 0.001, 0.024) (WtHR) β of ∑DEHP, 0.018 (95% CI:-0.001, 0.037) | sex. Age. residential area, average household income, physical exercise, and urinary creatinine level |
| 35 | J. On | 2021 | Urine of the participants at 5–16y | 5–16 y | Multivariate linear regression | β and 95% CI | (Weight percentile) β of MEHHP and MEOHP were 1.011 (95% CI: 0.530, 1.492), and -1.044 (-1.571, and -0.517) (Height percentile) β of MEHHP and MEOHP were 0.502 (95% CI: 0.001, 1.002), and -0.476 (-1.024, and 0.072) | age |
| 36 | C. Silva | 2021 | Urine of the participants at 6y | 6y and 10 y | Multivariate linear regression | β and 95% CI | (Fat mass index z-score, 6y) β of IQR increase of ∑DEHP, 0.10 (95% CI: -0.00, 0.20) (Fat mass index z-score, 10y) β of IQR increase of ∑DEHP, 0.09 (95% CI: -0.02, 0.21) | maternal educational level and child sex, age (except for sex- and age-adjusted BMI z scores), diet quality score, and television-watching time |
| 37 | E. E. Hatch | 2008 | Urine of the participants | 6–19y | Multivariate linear regression | β and 95% CI | (BMI) β of MEHHP (the highest quartile vs. the lowest quartile) among boys in 6–11 y and 12–19y were 0.42 (95% CI: -1.09, 1.92) and 1.00 (-0.69, 2.69), respectively (BMI) β of MEHHP (the highest quartile vs. the lowest quartile) among girls in 6–11 y and 12–19y were 0.54 (95% CI: -1.50, 2.57) and 0.74 (-1.18, 2.65), respectively (WC) β of MEHHP (the highest quartile vs. the lowest quartile) among boys in 6–11 y and 12–19y were 1.27 (95% CI: -2.43, 4.96) and 2.15 (-1.77, 6.08), respectively (WC) β of MEHHP (the highest quartile vs. the lowest quartile) among girls in 6–11 y and 12–19y were 1.83 (95% CI: -3.48, 7.13) and 3.14 (-2.41, 8.68), respectively | age, creatinine, height, race/ethnicity, socioeconomic status, % of daily calories from total fat, daily servings of dairy, daily servings of fruit and vegetables, METS/month, and TV/video/computer use |
| 38 | H. Wang | 2013 | Urine of the participants | 8–15y | Multivariate linear regression | β and 95% CI | (BMI) β of ∑DEHP, 0.037 (95% CI: 0.006, 0.067) (WC) β of ∑DEHP, 0.029 (95% CI: 0.001, 0.050) | age, sex, and urine specific gravity |

BMI, body mass index; OR, odds ratio; CI, confidence interval; CDGP, constitutional delay of growth and puberty; MEHHP, mono-(2-ethyl-5-hydroxy-hexyl) phthalate; MEOHP, mono-(2-ethyl-5-oxo-hexyl) phthalate; MECCP, Mono-2-ethyl-5-carboxypentyl phthalate; ∑DEHP, sum of di-2-ethylhexyl phthalate metabolites; MnBP, mono-n-butyl phthalate (MnBP); MBzP, Monobenzyl phthalate; WC, waist circumference; WHR, waist-to-hip ratio; WtHR, waist-to-height ratio; IQR, interquartile range
